# Organization from cell to tissue derived delivery systems for immunotherapy

**DOI:** 10.1016/j.mtbio.2026.103570

**Published:** 2026-08-14

**Authors:** Xi Tan, Mutang Li, Dandan Luo, Hongjun Li

**Affiliations:** a State Key Laboratory of Advanced Drug Delivery and Release Systems, School of Pharmacy, Zhejiang University, Hangzhou, China; b Jinhua Institute of Zhejiang University, Jinhua, China; c Department of Hepatobiliary and Pancreatic Surgery, The Second Affiliated Hospital, School of Medicine, Zhejiang University, Hangzhou, China

**Keywords:** Drug delivery, Biological platforms, Hierarchical organization, Immunotherapy

## Abstract

Dysregulation of the immune system drives a broad spectrum of diseases, including cancers, autoimmune disorders, and neuro-immune conditions. Immunotherapy has advanced from small-molecule drugs to biomacromolecular agents and living cell therapies, yet conventional synthetic delivery platforms struggle to preserve the structural integrity, bioactivity, and long-term viability of these complex payloads *in vivo*. Biologically derived drug delivery systems (BDDS) have emerged as a promising class of carriers that harness natural organelles, cells, and tissues to achieve high loading capacity, inherent biocompatibility, bioresponsive targeting, and controlled release. This review systematically examines the hierarchical organization of BDDS across nanoscale, microscale, and macroscale levels and emphasizes their distinct biological advantages, engineering features, and translational potential. We detail the material sourcing, isolation techniques, drug-loading, surface modification, and genetic engineering strategies, as well as considerations for scalable manufacturing. Representative platforms are highlighted for their ability to cross biological barriers, modulate immune microenvironment, and treat cancers, autoimmune diseases, and neuroinflammation. In addition, the emerging biosynthetic alternatives including membraneless organelle condensates, artificial cells, and advanced organoids, are discussed as next-generation platforms that may overcome the limitations of natural BDDS. Finally, we outline current challenges in the heterogeneity, large-scale production, and clinical translation, while proposing future directions including the reasonable administration routes, cross-scale hierarchical integrated platform designs and emerging computational and engineering technologies to accelerate the development of BDDS-based immunotherapies.

## Graphical abstract

**Figure:**
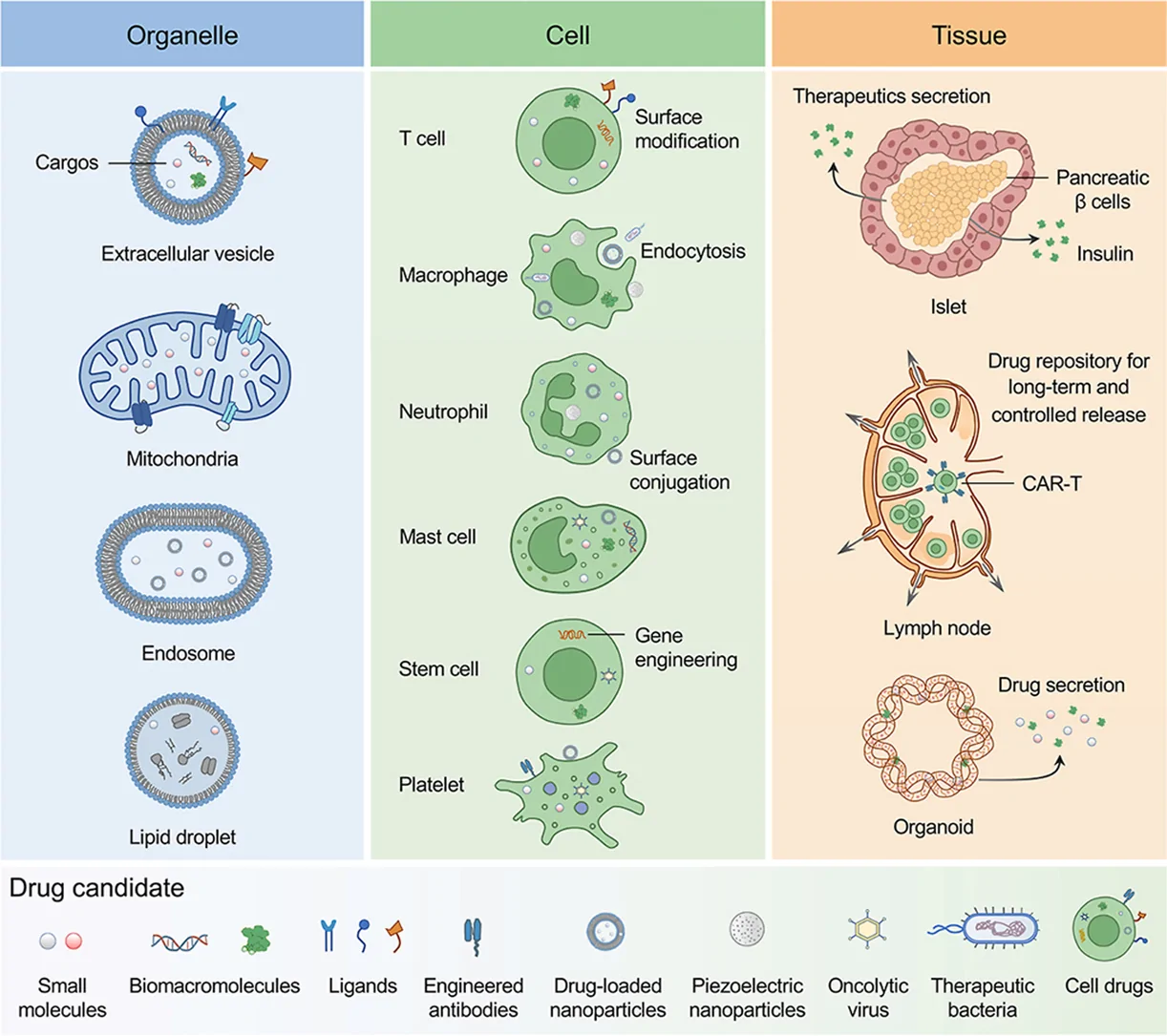

## 1 Introduction

Dysregulation of the immune system underlies a wide spectrum of devastating diseases, ranging from cancers to autoimmune disorders and neuro-immune conditions. These pathologies collectively affect hundreds of millions of individuals worldwide and represent a major unmet clinical need. Over the past three decades, immunotherapy has evolved dramatically from conventional small-molecule drugs such as dexamethasone and aspirin that merely relieve symptoms, to recombinant biologics including interleukin-2 (IL-2) and interferon-γ (IFN-γ) that provide more precise immune modulation, and ultimately to living cellular therapies such as chimeric antigen receptor (CAR)-T cells that hold the promise of functional cure [[1], [2], [3], [4], [5]]. Despite remarkable clinical successes, particularly in hematologic malignancies and an increasing number of solid tumors and refractory autoimmune diseases, the therapeutic potential of these advanced modalities remains constrained by delivery bottlenecks.

Conventional synthetic delivery platforms, including liposomes, polymeric nanoparticles, and microspheres, have proven effective for small-molecule drugs by enabling targeted accumulation and controlled release [[6], [7], [8]]. However, when applied to biomacromolecules such as proteins and nucleic acids, or living cells, these artificial systems encounter fundamental limitations. Importantly, these challenges vary substantially depending on the features of therapeutic cargos including small-molecule drugs, biomacromolecules, and living cell products. For small-molecule drugs, conventional formulations often suffer from poor aqueous solubility, rapid systemic clearance, limited tissue specificity, and dose-dependent toxicity [9,10]. Considering the biomacromolecular therapeutics, including proteins, nucleic acids, and gene-editing components, the additional barriers arise from their structural instability, enzymatic degradation, poor membrane permeability, and inefficient intracellular delivery [11,12]. As for living therapeutic cells, synthetic delivery approaches face even greater challenges due to the complexity of maintaining cellular viability, phenotype, and biological functions *in vivo* [13,14]. Peculiarly, engineered immune cells, stem cell-derived products, and other cellular therapies require preservation of metabolic activity, migration capacity, and functional stability after administration [15,16]. As a result, many promising immunotherapeutics suffer from poor bioavailability, rapid clearance, off-target toxicity, and suboptimal efficacy. These limitations highlight the necessity for smart delivery systems that intrinsically possess biological recognition, adaptive responsiveness, and functional integration with host tissues.

To overcome these critical challenges, researchers have increasingly explored biologically derived drug delivery systems (BDDS), which are therapeutic platforms derived from natural biological components or living systems, including organelles, cells, and tissues, and harness their intrinsic biological functions for targeted drug delivery and therapeutic intervention [17,18]. Unlike synthetic carriers, BDDS inherently preserve the structural integrity, bioactivity, and viability of complex payloads while leveraging endogenous biological features for superior performance. These platforms offer exceptionally high drug-loading capacity, excellent biocompatibility with minimal immunogenicity, intrinsic bioresponsive targeting such as inflammation chemotaxis and tumor-homing, efficient penetration across biological barriers, and precisely controlled release triggered by local biochemical or physical cues [[19], [20], [21]]. Furthermore, BDDS can actively participate in immune modulation by secreting bioactive factors and remodeling the local microenvironment, thereby achieving synergistic therapeutic effects that synthetic systems cannot replicate.

Previously, the existing reviews mainly focus on individual carrier categories, such as extracellular vesicles (EVs), engineered cells, or biomimetic nanoparticles [[21], [22], [23], [24], [25], [26], [27]]. In this review, we systematically examine the hierarchical organization of BDDS for immunotherapy, spanning nanoscale organelle-based platforms, microscale engineered cellular carriers, and macroscale tissue-derived vehicles. We discuss critical translational considerations including ethical and reproducible material sourcing, isolation and preservation techniques, versatile drug-loading and genetic-engineering strategies, and scalable manufacturing, while highlighting representative applications in cancer, autoimmune disorders, and neuro-immune conditions. We further explore emerging biosynthetic alternatives, such as membraneless organelle condensates, artificial cells, and advanced organoids, that build upon natural BDDS to combine biological fidelity with synthetic tunability. Finally, we critically assess current challenges and outline future directions to accelerate the clinical translation of these biologically inspired delivery platforms.

## 2 Design principles and engineering strategies of BDDS

**Fig. 1:**
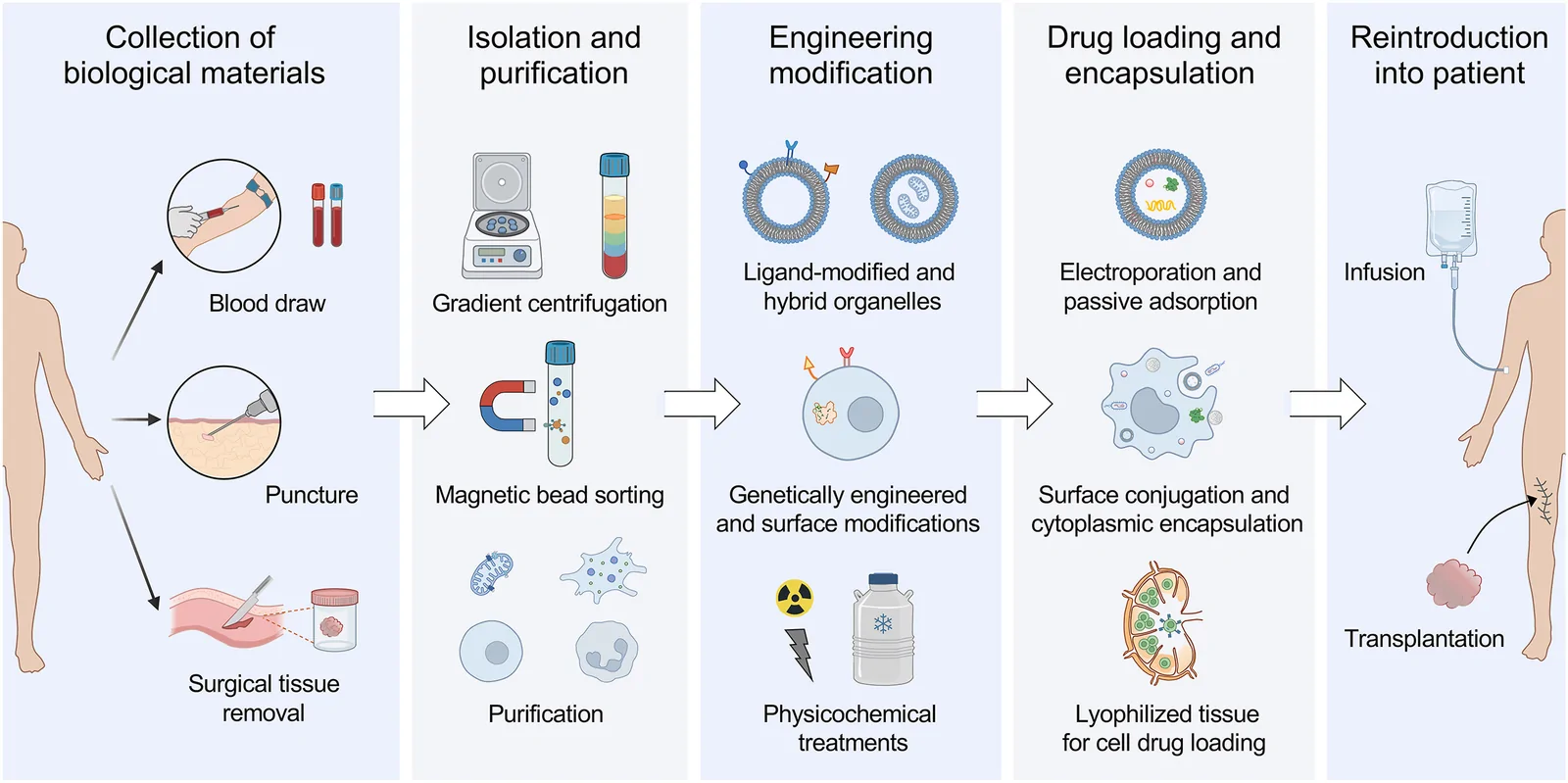
Schematic diagram showing the general process of BDDS preparation and its clinical applications. The process is mainly divided into the following steps: biological material collection, isolation and purification, engineering modification, drug loading and encapsulation, and reintroduction into patient.

The development of BDDS relies on diverse biological sources, including stem cells, immune cells, blood cells, organoids, and tissues, whose availability, ethical considerations, and intrinsic biological functions collectively determine their suitability for therapeutic applications. After the isolation and purification of biological materials, the drug loading can be achieved through electroporation, ultrasound, freeze-thaw, membrane insertion, and cell-drug conjugation, while surface modification and genetic engineering can enhance targeting, penetration, and responsive release of BDDS. The large-scale production depends on closed bioreactors and standardized cell expansion, with quality control focusing on functional activity, structural integrity, and biosafety. BDDS combine cell engineering, organoid technology, and strict quality control to provide a platform for clinically translatable precision drug delivery (Fig. 1).

### 2.1 Material sourcing, biocompatibility and biosafety considerations

A key limitation in the application of BDDS is the sourcing of biologically derived materials with ethical considerations that represents a significant challenge. Various cells isolated from human bone marrow, embryonic tissue, peripheral and umbilical cord blood, adipose tissue, and spleen tissue, have been widely employed in both preclinical research and clinical immunotherapy [28,29]. Among these, stem cells such as mesenchymal stem cells (MSCs) are typically obtained from adipose tissue, bone marrow, umbilical cord blood, placenta, or induced pluripotent stem cells (iPSCs). Their principal advantage lies in robust proliferative capacity. However, genetic engineering modifications remain technically challenging and carry potential risks of uncontrolled genomic alterations of stem cells [30]. In contrast, blood cells including red blood cells (RBCs) and platelets can be readily isolated from volunteer donors, offering minimal ethical concerns, low cost, and reduced potential risks *in vivo*. Immune cells represented by T cells, macrophages, and neutrophils, are generally obtained from bone marrow. Given the inherent differences in immunogenicity, autologous bone marrow-derived immune cells are preferred for engineered modifications and clinical immunotherapy, despite limitations in scalability and high cost. Collectively, these considerations underscore that the choice of cellular source is dictated by availability, ethical feasibility, and functional suitability for therapeutic application.

Beyond individual cells, tissue transplantation has long been applied clinically for disease treatment, including corneal transplantation for vision restoration, bone repair, and liver regeneration [31]. Autologous tissues offer minimal immune rejection and avoid ethical controversies, whereas allogeneic transplantation relies on voluntary donation, which is limited and carries inherent immunological risks. Consequently, autologous tissues represent ideal candidates for biologically derived cell-drug repositories and *in vivo* delivery platforms, offering advantages over biomimetic or synthetic materials such as hydrogels composed of polylactic acid (PLA), poly(lactic-co-glycolic acid) (PLGA), or hyaluronic acid. Nevertheless, both autologous and allogeneic approaches are constrained by donor availability and tissue matching. Researchers are also working on cultivating human organs in animals for clinical applications in organ transplantation with unknown immune rejection [32]. The choice between autologous and allogeneic sources represents a fundamental determinant of the translational potential of BDDS (Table 1). Although autologous platforms provide maximal biological compatibility, they face significant manufacturing and accessibility challenges. In contrast, allogeneic platforms offer scalability and “off-the-shelf” availability but require additional strategies to avoid immune rejection. From a clinical accessibility perspective, autologous BDDS remain limited by manufacturing turnaround time, specialized infrastructure requirements, and restricted treatment availability. Conversely, allogeneic platforms provide the potential for standardized, scalable, and rapid administration, making them more suitable for broad clinical implementation. Moreover, successful translation requires balancing manufacturing efficiency and costs, immune compatibility, biosafety, and long-term functional stability. As an alternative, stem cell-derived tissues generated *in vitro* provide a versatile strategy for both therapeutic purposes and as tissue-mimetic drug delivery platforms. Over the past decade, organoid technology has advanced rapidly, enabling the *in vitro* culture of tissue-like macroscopic structures that retain key functional properties of their native organs [33]. Organoid transplantation offers a promising alternative to conventional tissue grafts, mitigating issues related to tissue availability and ethical concerns. Certain organoids, including brain and tumor organoids, have now achieved commercially mature cultivation protocols, paving the way for their potential clinical application as tissue-like therapeutics or cell-based drug carriers to manage the ethical concerns and donor-sources of transplanted tissues.

**Table 1:** Comparative analysis of autologous and allogeneic BDDS.

Parameter	Autologous BDDS	Allogeneic BDDS
Sources	Patient-derived	Donor-derived
Immune rejection	Minimal	Moderate-high
Manufacturing	Individualized	Batch production
Cost	High	Lower after scaling
Production time	Weeks	Days-weeks
Quality variation	Patient-dependent	Donor-dependent
Clinical accessibility	Limited	High potential
Main challenges	Manufacturing complexity	Immune compatibility

To mitigate the potential pathogenic risks of live cell-based vectors, dead or dying cell-based delivery platforms also have been developed using diverse biological, physical, and chemical treatments for immunotherapy [34,35]. Liquid nitrogen-treated (LNT) cancer cells were proposed as targeted drug delivery vehicles and antitumor vaccines in combination with immune checkpoint blockade (ICB) antibodies [36]. UV-inactivated bacteria have been leveraged for photothermal immunotherapy across the blood-brain barrier (BBB), retaining intact cellular structures and chemotactic capabilities [37]. Enucleated MSCs and even cancer cells have been employed as carriers or therapeutic agents, preserving intrinsic functions such as secretion and migration while eliminating pathogenic risks [38]. Dying cancer cells (DCCs) were generated after the treatments with high concentrations of β-elemene (ELE), formulated in hydrogels (DCCs@ELE), as antitumor vaccines for chemo-immunotherapy against tumor recurrence [39]. Generally, the controllable fate of cells in the human body is a guarantee for the biosafety of cell-based carriers, cell drugs or tissue-like carriers.

Inactivated biological carriers, including tumor cell-derived carriers, enucleated MSCs, and inactivated bacterial platforms, represent an emerging class of “zombie cell” vectors that preserve selected biological functions while minimizing risks associated with living systems. Indeed, biosafety considerations are essential for evaluating inactivated cellular carriers. Residual pathogen-associated molecular patterns (PAMPs), such as lipopolysaccharide, lipoproteins, peptidoglycan, flagellin, and bacterial nucleic acids, as well as damage-associated molecular patterns (DAMPs) released during cell inactivation, may activate pattern-recognition receptors including Toll-like receptors and nucleotide-binding oligomerization domain (NOD)-like receptors, thereby triggering excessive innate immune activation and, in severe cases, cytokine storm. Therefore, the inactivation strategy should not only completely abolish proliferative or infectious potential but also minimize the exposure or biological activity of immunostimulatory components while preserving the desired delivery functions. However, their diverse origins and manufacturing processes require a unified biosafety evaluation framework to enable meaningful comparison and clinical translation. In our opinion, the biosafety assessment of inactivated biological carriers should include six major categories: residual biological activity such as viability, proliferation and metabolic activity, genetic and molecular safety without gene transfer potentials, immunological safety for controllable inflammatory activation, structural integrity and retained biological function to reduce potential toxicity caused by off-target delivery and drug leakage, and manufacturing and quality control associated with endotoxin levels and residual chemical or physical inactivation agents.

### 2.2 Isolation, purification, and preservation techniques

Advances in biological research have increasingly unraveled the complexity of cellular structures and functions. Techniques for organelle isolation and purification, as well as methods for preserving their biological functions, are now well-established, providing a solid technical foundation for employing biologically derived organelles as drug delivery vehicles [[40], [41], [42]]. Gradient centrifugation remains the most commonly used method for separating cells and organelles based on differences in shape, size, and density. In addition, a variety of alternative isolation techniques have been summarized in previous studies [24]. Currently, researchers can readily obtain commercial organelle/cell extraction kits to isolate and collect the abundant, functionally active organelles using standardized protocols. Furthermore, commercially available organelle/cell preservation solutions help maximize the retention of organelle activity and functionality, thereby supporting their application as *in vivo* drug delivery platforms. The antibody-labeled magnetic bead sorting method is sufficient to ensure the purity required for cell carriers and cell therapies [43]. Programmed cooling cryopreservation of cells maximizes the retention of cell viability and long-term usability. The development of serum-free cryopreservation solutions provides critical support for stem cell cryopreservation, avoiding the unfavorable cell differentiation during storage.

The clinical translation of BDDS requires not only therapeutic efficacy but also robust storage, transportation, and product stability strategies. Unlike normal synthetic platforms, BDDS contain complex biological components, including proteins, nucleic acids, membranes, and living cells, which are highly sensitive to temperature, osmotic stress, and structural disruption. For acellular platforms, including EVs, mitochondria, and organelle-derived carriers, the major preservation challenge is maintaining structural integrity, cargo stability, and biological activity during storage over months to years. Cryopreservation at low temperatures (−80 °C or liquid nitrogen conditions) is commonly used, with stabilizers such as trehalose, sucrose, albumin, and cryoprotective polymers employed to reduce membrane damage and aggregation. Recently, lyophilization has emerged as an attractive alternative because it enables room-temperature or refrigerated storage after drying, although optimization of rehydration protocols remains necessary to preserve vesicle morphology and functional activity. In contrast, living BDDS including engineered cell-derived products, organoids and tissue platforms, requires preservation approaches that can maintain a high cell survival rate, metabolic activity, and functional phenotype. Conventional cryopreservation typically relies on cryoprotective agents such as dimethyl sulfoxide combined with controlled-rate freezing and liquid nitrogen storage for months to years. However, prolonged storage may induce cellular stress, phenotypic drift, reduced proliferation capacity, or impaired therapeutic function. Considering tissue-like systems, additional challenges arise from maintaining three-dimensional (3D) architecture, extracellular matrix interactions, and multicellular organization during freezing and recovery. Therefore, cryopreservation agents for organoids and transplanted tissues are expected to sustain basic cellular physiological metabolism, possess high permeability and tissue penetration ability, be compatible with the preservation of multi-component cells, and maintain the spatial structure and morphology of the tissue as well as cell membrane stability, while avoiding ice crystal formation in the solution over days to months. At the same time, they should not contain components that may cause changes in gene expression, such as hormones (dexamethasone, prostaglandins, insulin), antibiotics (penicillin), and animal-derived proteins (bovine serum albumin, human serum albumin), thus solving the problem of fresh tissue sample preservation and transportation at moderately low temperature (4 °C) for hours to days. Commercially available preservation solutions include University of Wisconsin (UW) solution for active organ preservation during the clinical organ transplantation, advanced Hank's balanced salt solution (GLALA), HypoThermosol® FRS (BioLifeSolutions) as well as MACS Tissue Storage Solution for the tissue preservation in *ex vivo* experimental studies. Compared with cell-free carriers, living platforms generally require stricter cold-chain management and shorter transportation windows, whereas acellular carriers provide advantages in storage stability and logistical flexibility. Future development of BDDS products will require standardized preservation protocols, potency-based quality control assays, and scalable manufacturing processes compatible with GMP requirements.

### 2.3 Drug loading and encapsulation strategies

To facilitate the loading and targeted delivery of various therapeutics with a wide range of molecular weights, both physical, chemical and genetic engineering strategies have been extensively developed. Besides the most common passive diffusion or physical adsorption for drug loading, the most representative electroporation enables therapeutic proteins and nucleic acids to enter cell-based carriers through transient pore formation, and has been employed to load siRNA, mRNA or miRNA into vectors for treating cancer, neuroinflammation, and autoimmune disorders [[44], [45], [46], [47], [48]]. Physical methods such as ultrasound, freeze-thaw cycles, and nanopore perforation also facilitate macromolecular drug encapsulation [23,49]. Membrane insertion strategies allow hydrophilic drugs or functional proteins to be covalently linked to hydrophobic lipids or cholesterol, achieving surface modification with targeting ligands or monoclonal antibodies, as demonstrated in anti-PD-L1 engineered EVs with enhanced pharmacokinetics and antitumor efficacy [[50], [51], [52]]. However, physical drug-loading technologies for BDDS are still limited by low drug capacity, easy drug leakage, and the potential to damage the carrier's structure and affect its bioactivity.

Similar to antibody-drug conjugates (ADCs), chemical coupling is also introduced to build drug-carrier conjugates for BDDS preparation. Both organelle-drug conjugates and cell-drug conjugates (CDCs) provide a comprehensive strategy for loading small molecules, biological macromolecules, and nanomedicines on the surface of cellular vehicles [48,53]. Unlike drug loading into live cells, CDCs can better preserve the physicochemical properties of therapeutic agents while minimizing adverse effects on the physiological functions of carrier cells. Free drugs, nanoparticle-loaded therapeutics, or biological oncolytic viruses (OVs) delivered inside live cells, differ fundamentally from CDCs and must satisfy the following two key criteria. First, the therapeutic agent should exhibit minimal toxicity to the carrier cell, preserving normal cellular functions. Second, the carrier must possess robust uptake capacity, typically including phagocytic immune cells such as macrophages and neutrophils, or tumor cells with high endocytic activity. Structurally stable nanoparticles or biotherapeutics are generally internalized via charge-mediated or receptor-ligand interactions and then released through biologically responsive cell degranulation. For example, engineered immunoglobulin E (IgE)-sensitized mast cells (IgE-MCs) were designed to enable active targeting and tumor antigen-triggered degranulation-mediated OVs release for cancer immunotherapy [54]. Live cell carriers retain the ability to recognize and process endogenous biological signals as well as their own specific functions, which helps their targeted delivery to specific organs or lesions and subsequent responsive drug release.

BDDS including organelle and cell carriers, and tissue-like scaffolds can directly load small molecule drugs, whereas biomacromolecular drugs loading or *in situ* generation normally need to be implemented through genetically engineered technologies. Genetically engineered cells can efficiently produce and carry therapeutic proteins, nucleic acids, viruses, or bacteria. Especially, engineered cell carriers have clear advantages in smartly targeting tissues and lesions, carrying drugs for better penetration, prolonging drug effects, and site-specific releasing drugs. Lyophilized tissue as a new form of carrier, offers a sufficiently large and suitable loading space for macromolecular drugs and cell drugs, while also helping to preserve their biological activity and promoting the long-term survival of cell drugs [55,56]. In general, small molecules with low cytotoxicity and high affinity for cells in tissue-like carriers, especially immunotherapeutic drugs that require long-acting sustained-release applications, are highly compatible with tissue-based BDDS. Biologically derived tissue-like scaffolds are conducive to the recruitment, retention, and survival of target cells, making it possible to engineer cell-based drugs *in situ*.

### 2.4 Surface modification and functionalization

Surface modification of cell-derived vehicles mainly focuses on enhancing their tissue and lesion targeting ability, enabling the surface loading of therapeutic drugs or antibodies, improving the recognition and response capabilities of biological signals, as well as conferring characteristics that enable carriers to cross physiological barriers. Non-covalent modifications, including electrostatic interactions, biotin-avidin binding, and receptor-ligand recognition, provide alternative approaches, though they are generally less stable and may compromise organelle and cell surface properties [24,57]. Covalent coupling strategies, utilizing rapid and mild reactions such as N-hydroxysuccinimide-amine, maleimide-thiol, or azido-dibenzocyclooctyne chemistry, allow precise anchoring of drugs or functional proteins while preserving organelle integrity and activity [58,59]. Engineered EVs with high expression of targeting ligands have been demonstrated the improved organotropism and barrier penetration [60,61]. Biomimetic hybrid organelle systems combining EVs from multiple cell types, further enhance delivery efficiency, enable complex payload co-delivery, and correct immune dysfunctions across diverse disease contexts [62]. Mitochondria-enriched platforms such as super-EV-Mito, have demonstrated efficacy in rescuing mtDNA defects, alleviating tissue injury, and modulating immune responses [63]. These strategies position organelle-derived and hybrid organelle-based delivery systems as versatile and potent platforms for next-generation immunotherapy.

Cell carriers or cell drugs mainly consist of targeted modified antibody drugs, active factors that promote cell survival, and biomolecules that enhance tissue targeting and penetration ability. Modifications and functionalization on the cell surface mostly exist in the forms of CDCs. Moreover, the genetically engineered cells enable the consistent, scalable cell carriers or cell-based drugs that can be clinically translated into industrial applications. The genetically engineered surface modification and functionalization of cells are discussed in the next chapter.

### 2.5 Genetic engineering and synthetic biology approaches

Genetic engineering of parent cells enables the production of engineered organelles expressing specific proteins, enhancing drug loading, tissue targeting, and synergistic therapeutic effects. Engineered EVs with high expression of targeting ligands have been demonstrated to exhibit improved organotropism and barrier penetration [60,61]. Compared with organelles, cells possess more comprehensive biological functions, which can offer distinct advantages in drug loading capacity and versatility, targeted delivery, crossing of biological barriers, and responsive release at lesion sites. Moreover, cell carriers can themselves function as therapeutic agents to modulate and enhance immunotherapy. When selecting cell carriers for immunotherapy, the suitability of the cell type for the specific immune disease indication is critical. This selection leverages intrinsic properties such as cytokine-mediated chemotaxis, stem cell tumor-homing ability, inflammation-targeted migration, platelet-mediated aggregation in response to tissue injury, bioresponsive degranulation, and tumor-specific recognition by immune cells [27,64]. Among the most representative applications, genetically engineered CAR-T, CAR-macrophage (CAR-M), and CAR-natural killer (CAR-NK) cell therapies serve both as cellular carriers and as cell-based therapeutics, exemplifying the dual role of engineered cells in precision immunotherapy.

Genetically engineered cell carriers primarily aim to enhance tissue targeting and penetration. Such platforms are designed for controllable *in situ* biosynthesis and release of biomacromolecular drugs at lesion sites, responsive to biological, physical, or chemical stimuli. CD19-targeted CAR-T cells exemplify genetically engineered tumor-targeted cells, serving simultaneously as vehicles for enhanced OVs delivery in cancer immunotherapy [65]. Despite their efficacy, CAR-T therapies face clinical challenges related to overactivation and limited control of cytotoxicity. To address this, a resveratrol (RES)-responsive transactivator and transrepressor system was developed to fine-tune CAR expression and control T cell cytotoxicity [66]. Similarly, engineered OVs were generated to amplify viral release from infected tumor cells via cell lysis, enhancing oncolysis through ultrasound (US)-triggered expression of the N-terminal domain of gasdermin D (GSDMD^NT^) *in vivo* [67]. Genetically engineered cell carriers can be tailored to achieve controllable activity, precise drug release, enhanced therapeutic efficacy, and improved biosafety.

Genetic engineering substantially enhances the therapeutic functionality of BDDS, whereas the engineering process itself may compromise product quality and biological activity. There are two major challenges including transfection-associated cytotoxicity and phenotypic drift of stem cells such as iPSCs during prolonged *in vitro* expansion in genetic engineering BDDS for immunotherapy. Transfection-induced membrane damage, oxidative stress, mitochondrial dysfunction, and transient inflammatory responses, will compromise cell viability, proliferative capacity, and cellular function, thereby diminishing the therapeutic efficacy of both the engineered cells and their derived platforms. Extensive cell expansion during the manufacturing of cell products, may alter gene expression, epigenetic status, metabolic activity, and differentiation potential. Thus, manufacturing strategies should prioritize minimally perturbative engineering approaches, including transient mRNA delivery, optimized non-viral transfection technologies, and genome-editing methods with reduced cellular stress. In parallel, standardized culture protocols, early-passage cell banking, continuous phenotypic monitoring, and potency-based release assays should be incorporated into quality control workflows to preserve biological function and ensure manufacturing reproducibility.

### 2.6 Scalable manufacturing and quality control

Given the substantial number of cellular carriers required for therapeutic purposes, scalable proliferation and *in vitro* expansion of human cells constitute another critical issue for clinical translation. From the perspective of rapid and efficient amplification production, HEK293 and CHO cell lines approved by FDA for viral vaccines or recombinant protein vaccines are the preferred choice for scaled-up production and isolation of organelles. HEK293 cell lines, leveraging the advantages of humanized post-translational modifications, produce protein drugs that are more in line with human physiological characteristics and have lower immunogenicity. Human-derived stem cells and iPSCs exhibit better biocompatibility than the mouse-derived production cells while ensuring amplification and production efficiency. The main issues faced by stem cell therapy are the potential carcinogenic risks in the body and the difficulty of engineering modifications. Notably, stem cells offer rich and young mitochondrial sources for the injury regeneration repair, and anti-aging applications represented by mitochondrial transplantation [68,69]. Engineered immune cell therapies, represented by engineered CAR-T cells, are limited by low gene transfection efficiency, expansion efficiency, and long-period production, resulting in high clinical treatment costs. The closed bioreactors for cell suspension culture, supplemented with automated monitoring, are the main technologies for large-scale cell production. Currently, human-origin organs and tissues available for transplantation cannot be produced on a large scale. Scientists are exploring the cultivation of humanized organs using genetically engineered experimental pigs for organ transplantation [70]. Similarly, organoids also face bottlenecks in scaled-up trial production. Current organoid fabrication often relies on labor-intensive protocols, animal-derived extracellular matrices, and poorly standardized differentiation processes, making large-scale manufacturing challenging. Future efforts should focus on chemically defined culture systems, automated bioprocessing, closed manufacturing platforms, and real-time quality monitoring to enable scalable, reproducible, and clinically compliant organoid production.

Actually, the quality control of biologically derived materials applied for drug delivery platforms, is the most critical challenge restricting clinical translation. Batch processing is the first link that restricts BDDS production. The active functional state of organelles and cells depends on the current survival state of cell sources, affecting their pharmacokinetic processes and fate in the body. Furthermore, organoids are influenced by numerous factors such as the source of iPSCs, the uncontrollable and long culture periods, and varying dimensions in shape and size. Apart from the microbial contamination, endotoxins, and virus detection directly related to biosafety, organelles mainly focus on size, protein levels, key protein drug content, and activity status. In particular, the density and composition of membrane proteins and other surface-associated components, as well as the colloidal stability of cell-derived platforms, critically determine their performance as delivery vehicles by regulating cellular recognition, tissue homing, circulation time, and immune evasion, thereby shaping their pharmacokinetics, biodistribution, and therapeutic efficacy. The normal quality control of cell-based products mainly focuses on the functional indicators such as the killing activity of CAR-T cells, cell phenotype, proliferative activity, cell differentiation, and genetic stability. In comparison, organoid quality control pays more attention to its structural integrity including organizational hierarchy, cell distribution via immunostaining assay and tissue sections. Humanized organs or tissues cultivated in different species usually need to consider tissue rejection, genetic contamination, and potential systemic diseases after the transplantation into the body. To minimize donor-to-donor and batch-to-batch variability, standardized donor selection criteria, chemically defined culture conditions, quantitative proteomic characterization, and potency-based release assays will be essential for ensuring product consistency and facilitating the clinical translation of BDDS.

## 3 Hierarchical organization of BDDS

**Fig. 2:**
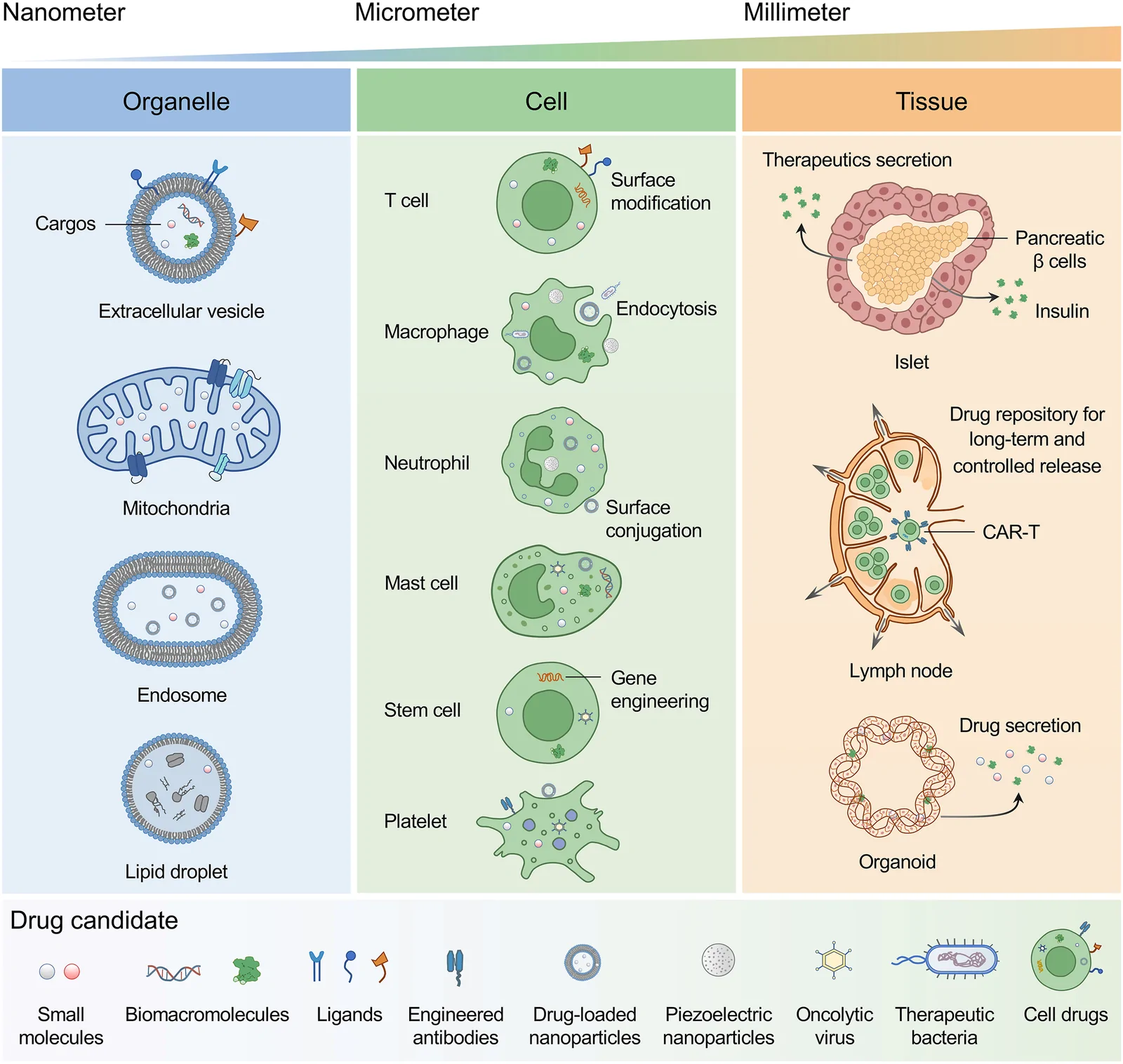
**Organization from cell to tissue applied for BDDS.** Nanoscale (10∼1000 nm) organelles, microscale (1∼50 μm) cells, and macroscale (>50 μm) tissues are engineered as vectors for delivering various drugs including small molecular drugs, therapeutic biomacromolecules, functional ligands, antibodies, drug-loaded nanoparticles, piezoelectric nanoparticles, oncolytic virus, as well as engineered therapeutic bacteria and cells. Organelles such as extracellular vesicle, mitochondria, endosome and lipid droplet, usually serve as the nanoscale vehicles with good biocompatibility for small molecular and macromolecular drugs via the internal loading or surface modification. Immune cells such as T cells, macrophages, neutrophils, mast cells, and stem cells such as MSCs and neural stem cells (NSCs) as well as blood cells such as platelets, are engineered as microscale vectors to load small molecules, therapeutic protein and nanomedicine for tissue-targeted delivery across biological barriers. Macroscale tissue-derived platforms represented by the transplanted islet, lyophilized lymph node tissue and biosynthetic organoid, can serving as a living pharmacy to *in situ* produce bioactive insulin, as a reservoir and delivery vehicle for CAR-T cells or other therapeutic products.

The human body exhibits a hierarchical organization, from organs to tissues, cells, and finally to the subcellular level represented by organelles. This size-based organization ensures that bioactive small molecules, macromolecules, and cells can function optimally within the organism. Correspondingly, therapeutic interventions must engage the body at appropriate dimensional scales to achieve their desired effects. Immunotherapeutic agents span this spectrum, from small-molecule immunoregulatory drugs with molecular weights of several hundred daltons (Da), to macromolecular biologics including protein and nucleic acid drugs ranging from tens to hundreds of kilodaltons (kDa), and extending to micrometer-scale therapeutic cell products, exemplified by engineered CAR-T cells. Accordingly, the design of biological delivery systems is guided by size: nanoscale (10∼1000 nm) organelles, microscale (1∼50 μm) cells, and macroscale (>50 μm) tissues are leveraged as carriers to match the dimensions of their respective therapeutic payloads *in vivo* (Fig. 2). Although BDDS possess unique biological advantages, quantitative comparison among different platforms remains challenging due to variations in preparation methods, cargo types, administration routes, animal models, and analytical approaches. Nevertheless, establishing standardized benchmarking parameters is essential for evaluating translational potential and guiding platform selection. To address this issue, we have summarized representative quantitative metrics from recent studies, including payload loading capacity, particle size, circulation half-life, biodistribution profiles, and immunomodulatory outcomes (Table 2). These comparisons reveal distinct performance characteristics among different BDDS categories. For example, EVs-based systems generally exhibit favorable biocompatibility and nanoscale tissue penetration, whereas engineered cellular platforms provide superior cargo capacity and sustained biological activity but face greater manufacturing complexity. Membrane-derived carriers demonstrate advantages in immune evasion and targeting through endogenous surface proteins, while organelle-based systems offer unique opportunities for regulating intracellular metabolism and immune signaling.

**Table 2:** Quantitative comparison of representative BDDS for immunotherapy.

Platforms	Size range (μm)	Payload capacity	Administration routes	Circulation or persistence time	Biodistribution	Major immunomodulatory effects	Main limitations
Organelle	0.01 ∼ 1	Molecular drugs	Systemic and local administration	Minutes-hours to days	Specific tissue-targeted	DC maturation, T-cell activation	Low yield, heterogeneity
Cell	1 ∼ 50	Biomacromolecular therapeutics	Systemic administration and local implantation	Hours to days	Specific tissue-targeted	Direct immune activation/killing	Phenotypic mutation and tumorigenicity
Tissue	> 50	Cell drugs	Local implantation	Days to months	Local rentention	Immune microenvironment remodeling	Manufacturing complexity

### 3.1 Nanoscale: organelle-derived platforms

Organelles, as the primary structural and functional units of cells, play a pivotal role in maintaining normal cellular activities and specialized functions. In recent years, biomimetic drug delivery systems have evolved from using biologically derived lipids and proteins to exploiting whole organelles, which retain more complete biological functions, including signal transduction pathways that initiate downstream responses for immunoregulatory effects, beyond their role as mere carriers [[71], [72], [73]]. Among these, EVs are integral to immune system communication, have been widely employed for targeted drug delivery in cancer immunotherapy and autoimmune disorders. Apart from EVs, the intracellular endososomes, mitochondria, lipid droplets (LDs) and other membraneless compartments also have been explored as vehicles for the specific immune dysfunction and cancer immunotherapy. Similar to human-derived organelles, organelles isolated from animals and plants also exhibit significant potential for therapeutic applications across a range of immunologically relevant diseases.

Organelles generally preserve cell-specific molecular signatures and biological functions inherited from their parent cells, thereby influencing their potential as targeted drug delivery carriers (Table 3). For instance, EVs and exosomes derived from platelets or immune cells confer both site-specific targeting such as inflamed or damaged vasculature and immunoregulatory capabilities. Consequently, the cell type determines the applicability of organelles in immunotherapy, guiding their delivery across biological barriers. Even organelles from the same cell type but different tissue origins exhibit variability in protein and miRNA content, affecting key biological processes including cell migration, proliferation, differentiation, and immune modulation. Furthermore, the age and bioactivity of the source cells can change the targeting and immune-regulatory performance of organelle carriers. The physicochemical properties, molecular compositions, and biological functions of isolated organelles are strongly influenced by donor age, tissue origin, and cellular state, which may ultimately affect targeting efficiency, therapeutic efficacy, and manufacturing reproducibility. Collectively, the cellular and tissue origins of organelle-based carriers constitute a decisive factor limiting their therapeutic applications.

**Table 3:** Organelle-derived nanoscale BDDS for immunotherapy.

Carriers	Sources (Species/cell types)	Therapeutic cargos	Loading methods or engineering strategies	Main characteristics	Mechanisms of action	Therapeutic applications in disease	Limitations	Clinical stage	References
**EVs**	Platelets (animal)	NLRP3-inflammasome inhibitor MCC950	Hydrophobic interaction	The inherent affinity for inflammation sites	Blocking NLRP3 activator- mediating IL-1β release	Atherosclerosis	Scale production and quality control	Preclinical stage	[74]
		TPCA-1	Electrostatic and hydrophobic interactions	The inherent affinity for inflammation sites	Inhibition for the production of inflammatory factors	Pneumonia	Low drug loading and quality control	Preclinical stage	[75]
		TLR agonists R848	Sonication and freeze-thaw	The selective adherence to tumor cells or tumor-related cells	TLR agonists for the activation of antitumor responses	Tumor	Quality control	Preclinical stage	[76]
	CAR-T cells (human)	Cetuximab and trastuzumab	Genetic engineering modification	Carrying CAR on their surface	Direct immunosuppression and immune killing effects	Tumor	Scale production and quality control	Preclinical stage	[77]
	CAR-NK cells (human)	CD133 nanobody	Genetic engineering modification	Cell-free therapeutic platform targeting tumor	Enhanced infiltration and activation of polarized macrophage and neutrophil	Tumor	Low yield and scale production	Preclinical stage	[78]
	Macrophages (animal)	miR-709	IL-4 for polarization of the parent macrophages	miR-709 transferred into alveolar macrophages	Inhibition of the NF-κB/NLRP3 inflammasome axis	ALI	Low drug loading	Preclinical stage	[79]
	Microglia (animal)	Inherent bioactive components	IL-4 for polarization of the parent microglia	Retinal microvascular remodeling	Facilitation of polarized M2 retinal microglia	Diabetic retinopathy	Scale production	Preclinical stage	[80]
	MSCs (human)	Phosphatase and tensin homolog siRNA	Electroporation	Chemotaxis migration and high affinity to neurons	Reduction of microgliosis and astrogliosis and promotion of axonal growth and neovascularization	Spinal cord injury	Low drug loading	Preclinical stage	[81]
	Macrophages/MSCs (animal)	NAD(P)H mimic (MitoN)	Co-extrusions for membrane fusion	Inflammation targeting and the cardiac stroma penetration	ROS scavenger to alleviate oxidative damage of cardiomyocyte	Myocardial injury and myocarditis	Complicated preparation process and quality control	Preclinical stage	[62]
	NSCs (animal)	Nano-selenium	*In situ* nucleation growth	BBB penetration capacity	ROS scavenging and neuroprotection	Traumatic brain injury	Quality control	Preclinical stage	[82]
	Cancer cells (animal)	Succinate	Electroporation	Homing effect and efficient uptake by macrophages	Polarization of TAMs via metabolism reprogramming	Tumor	Potential safety risk	Preclinical stage	[83]
		Inherent bioactive components	Pyroptosis induced by lipopolysaccharides (LPS)	Vaccine	Activation of antitumor immune response	Tumor	Quality control	Preclinical stage	[84]
	Milk (animal)	Inherent bioactive components	Differential ultracentrifugation	Containing abundant immune-active proteins	Regulation of host intestinal immune homeostasis and gut microbiota	Ulcerative colitis	Quality control	Preclinical stage	[85]
	Spinach (plant)	Inherent bioactive components	Folate-modification	Generation of oxygen and NADPH	Macrophages polarized from M1 to M2	Rheumatoid arthritis	Quality control	Preclinical stage	[86]
	Young blood (animal)	Inherent bioactive components	Size-exclusion chromatography	Abundant Klotho mRNA within young EVs transferred to recipient cells	Recovery of aged cell bioenergetics and regeneration of skeletal muscle	Anti-aging	Quality control	Preclinical stage	[87]
**Mitochondria-containing EVs**	Skeletal muscles (animal)	Intact mitochondria	Differential ultracentrifugation	Mitochondrial genome transfer for enhanced mitochondrial biogenesis	Rescuing mitochondrial damages and alleviating its associated inflammation	Muscle in jury and kidney injury	Complicated preparation process and quality control	Preclinical stage	[88]
**Mitochondria**	Cardiomyocyte (animal)	Intact mitochondria	Sonication for the activated neutrophil membrane-fusion	Organ injury targeting ability, healthy mitochondria transfer via tunneling nanotubes (TNTs)	Anti-inflammation and cell repair	Acute organ injuries	Low delivery efficiency	Preclinical stage	[89]
	Cancer and cancer-related cells (animal)	Nanoparticles containing SPION and IR780	Electrostatic and hydrophobic interactions mediated by IR780	Drug-loaded mitochondria transfer via TNTs	Photodynamic killing effects	Tumor	Complicated preparation process and quality control	Preclinical stage	[90]
	Cardiomyocyte (animal)	Fused peptide SDKP-SS31	Mitochondrial-targeted binding property of SS-31	Reduced neutrophil extracellular traps (NETs) formation	Alleviation of inflammatory and oxidative stress, protection from cardiomyocytes apoptosis	Myocarditis	Low targeted delivery efficiency and potential off-target	Preclinical stage	[91]
	Cancer cells (animal)	OVA/TRP2	Genetic engineering modification	Antigens stimulants for DCs-targeted delivery	Activation of the adaptive cellular immunity	Tumor	PotentialSafety risk	Preclinical stage	[92]
**Endosomes/lysosomes**	Cancer cells (human)	Doxorubicin (DOX)	Magnetic extraction	Specific homotypic affinity	Chemotherapeutic effect	Tumor	Low yield	Preclinical stage	[93]
	*Saccharomyces cerevisiae* (Fungi)	HER2-specific affibody and DOX	Genetic engineering modification	Cancer-targeting and enhanced penetratin	HER2 signaling, activating the immune system and chemotherapy	Tumor	Quality control	Preclinical stage	[94]
**LDs**	Safflower seed (plant)	Oleosin-rhFGF9 fusion protein	Transgenic plant	High efficient absorption and penetration	Growth factors promoting regeneration and repair	Hair loss and skin wound	Immune stimulation	Preclinical stage	[95]
	Group 2 innate lymphoids (ILC2s) (animal)	HMGB1	Genetic engineering modification	Intercellular delivery of signals mediated by LDs	Activation and infiltration of neutrophils	Allergic inflammation	Difficult to separate and low yield	Preclinical stage	[96]

Platelet-derived EVs (PEVs) actively target damaged vessels and activated endothelial cells, mediating inflammatory responses via platelet-specific proteins. Platelet membrane-derived nanovehicles (PM-NVs) were first developed to mimick platelets, which both carry the active protein and small-molecule chemotherapeutic agent for tumor-targeted sequential delivery and specific elimination of circulating tumor cells (CTCs) [97]. A NOD-like receptor thermal protein domain associated protein 3 (NLRP3)-inflammasome inhibitor MCC950-loaded PEVs was developed for atherosclerosis-targeted therapy [74]. Engineered PEVs were also applied to deliver the anti-inflammatory dr[75]ug TPCA-1 to the lungs, significantly reducing pulmonary inflammatory cell infiltration and alleviating cytokine storm syndromes [75]. Similarly, platelet membrane-cloaked nanoparticles (PNPs) were generated for tumor-targeted delivering Toll-like receptor agonists R848 to elicit antitumor immune responses [76]. A major advantage of platelets is their ability, upon activation, to generate abundant vesicle-like particles, providing a rich source of delivery carriers. Moreover, in clinical practice, platelets can be isolated from allogeneic blood during standard blood processing, addressing cell source limitations, while platelet-derived vesicles overcome the challenges associated with platelet storage.

Immune cell-derived EVs generally retain the targeting characteristics mediated by the specific recognition of damaged cells at inflammatory sites or tumor cells. CAR-T cell-derived exosomes carrying surface CAR molecules can directly replace CAR-T cells in cancer immunotherapy, exhibiting potent antitumor activity with reduced toxicity [77]. CAR-NK vesicles were generated from CAR-engineered iPSCs for tumor targeting and enhancing host innate immunity for cell-free immunotherapy [78]. M2-like macrophage-derived EVs (MEVs) could mitigate acute lung injury (ALI) induced by lipopolysaccharide via intrinsic miR-709 activity [79]. M2 microglia-derived exosomes suppress neuroinflammation and promote neurite growth, serving as trans-BBB delivery carriers for brain injury and ischemic stroke [80,98,99]. The activation or polarization state of immune cells profoundly influences their protein expression and functional properties. Accordingly, the selection of cell-derived organelles should consider the immune cells’ functional state, or preconditioning strategies, such as cytokine stimulation, should be employed.

Stem cell-derived EVs carry abundant cytokines and bioactive molecules that facilitate tissue repair. MSC-derived EVs (MSC-EVs) replicate MSC therapeutic benefits, serving as delivery carriers for spinal cord injury by modulating the neuroinflammatory microenvironment [81]. NSC-derived EVs (NSC-EVs) inherit neuroprotective functions, delivering non-coding RNAs, proteins, lipids, and other bioactive components to target cells. Functionalization with ultrasmall nano-selenium enables NSC-EVs to cross the BBB via APOE/LRP-1 interactions, scavenging ROS and reducing traumatic brain and spinal cord injuries [82]. Nasal administration of stem cell-derived EVs has demonstrated rapid brain delivery in clinical trials, alleviating neuroinflammation and neurodegenerative disease symptoms while restoring cognitive function [100,101]. Stem cell-derived vesicles are anticipated to provide a safer and more efficacious alternative, with the potential to replace stem cell therapy in immunotherapeutic applications.

Tumor cell-derived EVs (TEVs) display tumor homing and high affinity for tumor cells, supporting their use as targeted delivery vehicles. TEVs localize to their parent tumor cells and exhibit organ-, tissue-, and cell-specific tropism of tumor lesions [[102], [103], [104]]. Leveraging their uptake by macrophages, TEVs have been applied to reprogram tumor-associated M2 macrophages to M1 phenotypes, as exemplified by succinate-loaded TEVs for antitumor immunotherapy [83]. Physicochemical processing further modulates organelle composition and function. For example, pyroptotic vesicles derived from LPS- and nigericin-treated cancer cells exhibit abundant tumor antigens and strong immunostimulatory effects [84]. Obtaining sufficient quantities of highly pure TEVs from tumor cell culture supernatants or patient body fluids remains a significant challenge. Tumor cells secrete only limited amounts, which are frequently contaminated with cell debris, apoptotic bodies, lipoproteins, and virus-like particles. Methods that achieve high purity typically result in low yield, limiting their suitability for large-scale drug loading or vaccine production.

**Fig. 3:**
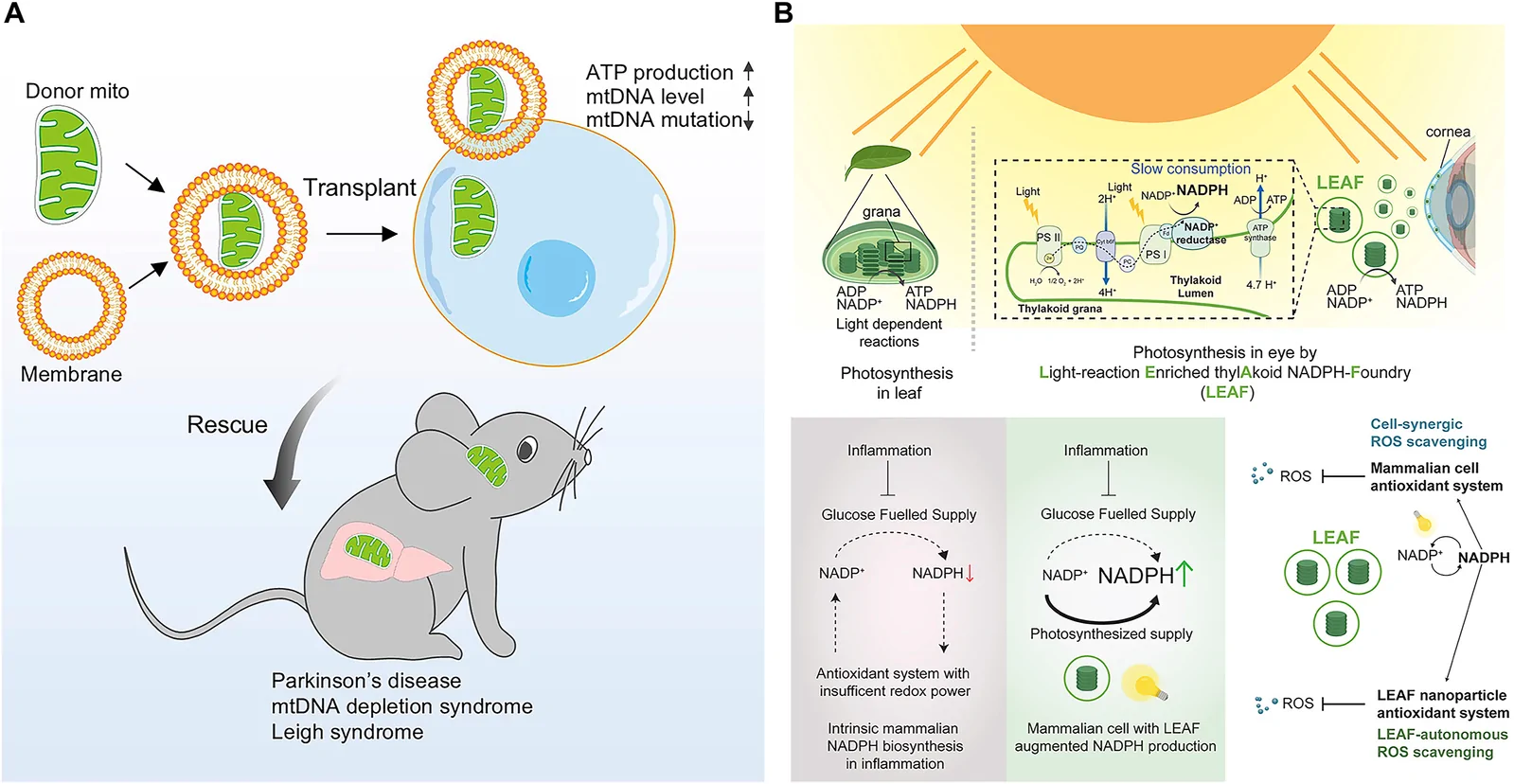
Subcellular structure-derived nanoscale platforms transplantation for immunotherapy. (A) Mitochondria isolated from donor cells, is encapsultaed by erythrocyte membranes as mitochondrial capsules to rescue PD, mtDNA depletion syndrome and LS following systematic delivery. *Reproduced with permission. Copyright 2026, Cell* [105]. (B) Plant leaf-derived thylakoid grana is encapsulated by surfactant Pluronic F127 to generate the nanoscale light-reaction enriched thylakoid NADPH-foundry (LEAF), which is then delivered into corneal cells to release NADPH and eliminate ROS by the *in situ* photosynthesis for the chronic inflammation-mediated keratoconjunctivitis sicca (KS) therapy. *Reproduced with permission. Copyright 2026, Cell* [106].

Mitochondria derived from stem cells or skeletal muscle cells confer resistance to oxidative stress, enhance cell survival, and promote tissue repair. Beyond serving as carriers, mitochondria actively participate in immune regulation, with potential applications in skin, cardiac, and brain injury therapies. Engineered mitochondria as delivery vehicles or therapeutic drugs were also developed to alleviate myocarditis, autoimmune and inflammatory diseases [90,91]. Engineered mitochondria, including OVA-MITO and TRP2-MITO from genetically modified tumor cells, also serve as antitumor vaccines [92]. The primary limitation of mitochondrial transplantation and the application of mitochondria as drug delivery vehicles lies in their low cellular uptake efficiency. To overcome the biological barriers for *in vivo* systemic delivery and transplantation of mitochondria, mitochondria encapsulated within erythrocyte membranes have been developed, providing a strategy to mitigate immune-related diseases such as Parkinson's disease (PD) and Leigh syndrome (LS) arising from the mitochondrial dysfunction (Fig. 3A) [105]. Hybrid nMITO platforms were generated by the fusion of neutrophil membranes with autologous muscle-derived mitochondria, exploiting cell membrane targeting and mitochondrial anti-inflammatory functions for acute organ injury treatment [89]. Encapsulated mitochondria and intercellular mitochondrial transfer via TNTs, facilitate targeted delivery and deep tissue penetration of mitochondria-based therapeutics.

Besides the above common organelles, other organelles including LDs, endosomes/lysosomes, peroxisomes and even endoplasmic reticulum (ER)-derived vesicles were also explored as the carriers or biotherapeutics for immunomodulatory treatments. LDs regulate immune cell functions beyond intracellular fat storage. Extracellular LDs released by ILC2 cells could transport alarmins such as HMGB1 to activate neutrophils and mediate allergic inflammation [96]. Considering high yield of LDs drived from plants, researchers have used transgenic plants to extract and isolate the engineered LDs containing therapeutic FGF9 fusion proteins, to promote hair regrowth and heal skin damage [95]. Endogenous membrane-bound organelles inside cells, like endosomes/lysosomes, are similar to EVs and have a very high similarity to the cell membrane in terms of protein and lipid composition. Engineered lysosomes were isolated from the engineered cells by magnetic extraction or differential ultracentrifugation, as molecualr drug vehicles or therapeutic proteins intergrated platforms with specific homotypic affinity for antitumor treatments [93,94]. Apart from lysosomes, small intracellular vesicles derived from multiple organelles including ER, Golgi apparatus or endosomes within MSCs from umbilical cord, were also isolated as promising candidates for delivering the inherently bioactive molecules and therapeutic compounds to the diseased retina [107]. EM membrane-intergrated hybrid lipoplexes were developed to alter internalized process and facilitate cytoplasmic delivery of epidermal growth factor receptor (EGFR)-targeted siRNA for antitumor therapy [108]. The proteasome is involved in regulating redox balance and inflammatory responses through reactive oxygen (ROS) modulation. However, it is still a challenge for collect the enough and purified proteasomes as vehicles with original biological activity for immunotherapy. Given challenges in separation, purification, preservation, and cargo loading of these underrepresented organelles with relatively low yield, the artificial biometric organelle-like structures are being explored as potential immunotherapy vectors for biomacromolecule therapeutics.

Organelle sources are expanding beyond human cells to include animal- and plant-derived materials. Milk-derived EVs modulate intestinal immune homeostasis and microbiota, alleviating colonic inflammation [85]. Nanosized LEAF was developed from plant leaf-derived thylakoid grana, to generate NADPH and ATP *in situ* for the clearance of ROS used for the treatment of KS caused by the corneal cell oxidative stress and chronic inflammation (Fig. 3B) [106]. Engineered spinach-derived thylakoids (FA-PEG-NTK) leverage folate receptor-targeted photosynthesis to generate oxygen and NADPH, reprogramming macrophages toward an anti-inflammatory M2 phenotype for rheumatoid arthritis therapy [86]. Organelle functionality also depends on tissue origin, cellular state, and age. Donor age has been associated with age-dependent alterations in mitochondrial bioenergetics, lysosomal function, and intracellular redox homeostasis, potentially reducing the therapeutic potency of organelle-based products.

Senescent donor cells often exhibit altered expression of inflammatory mediators, extracellular matrix-associated proteins, and stress-response pathways, potentially affecting the biological performance of cell-derived carriers. In contrast, EVs from young murine serum restore aged cell bioenergetics and promote muscle regeneration, whereas EVs from aged donors may impair recipient cells [87]. Similar results have also been verified in the application of young mitochondria in accordance with the differences in proteomics and metabolomics [109,110]. Moreover, donor inflammatory status can markedly alter the cargo profile of biological carriers. Comparative miRNA profiling has demonstrated that EVs derived from inflammatory or disease-associated cells contain distinct miRNA signatures involved in immune activation, macrophage polarization, and cytokine regulation [[111], [112], [113]]. Such inflammation-associated molecular remodeling may enhance or impair therapeutic effects depending on the intended application. Likewise, organelles isolated from different tissues exhibit distinct proteomic, lipidomic, and metabolic profiles that reflect tissue-specific physiological functions, leading to differences in biodistribution, cellular uptake, and immunomodulatory capacity. MSC-EVs from different tissue sources vary in bioactive contents such as functional proteins and miRNA according to the comparative proteomic and transcriptomic analysis, influencing anti-inflammatory signaling, cell growth, and tissue repair [114,115]. Furthermore, the physiological state of donor cells including inflammatory activation, hypoxia, senescence, metabolic stress, or disease conditions, can substantially reshape organelle composition and function, thereby altering their interactions with recipient cells. Overall, selecting the appropriate cell type, source age, and modification strategy is critical for optimizing organelle-based drug delivery systems in immunotherapy.

### 3.2 Microscale: engineered cell-based platforms

**Fig. 4:**
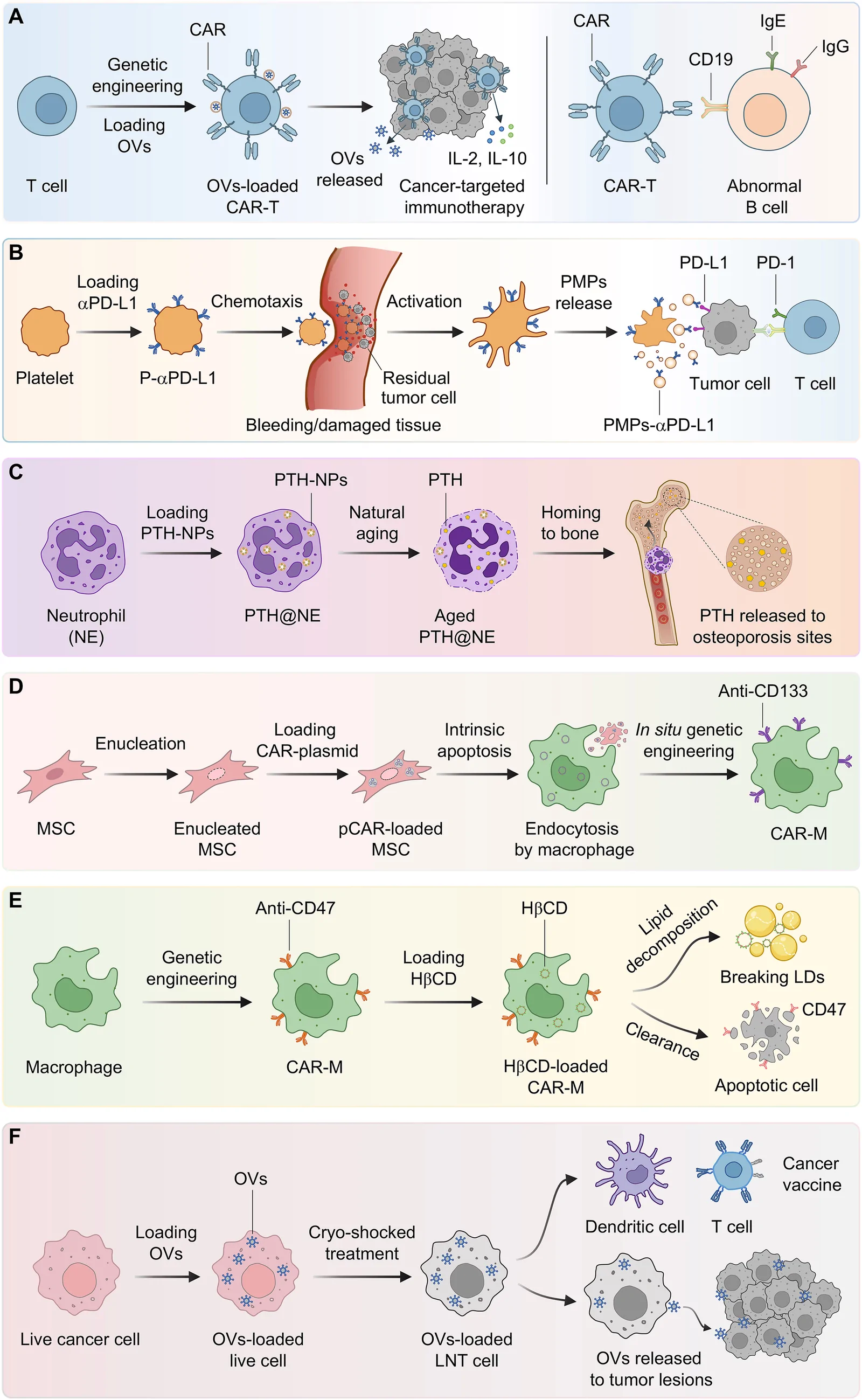
Engineered cells as drug delivery vehicles or therapeutic cell products for immunotherapy. (A) Engineered CAR-T cells for tumor-targeted delivery and release of OVs followed by cytokine extraction to enhance cytotoxicity or enable abnormal B cell depletion for immunotherapy. (B) Engineered platelets targeting the bleeding/damaged tissues are activated to release αPD-1 in the PMPs for post-surgical cancer recurrence immunotherapy. (C) Aged neutrophils homing to the bone marrow facilitates the targeted delivery and boosted release of bioactive peptide drugs for osteoporosis immunotherapy. (D) Macrophage-specific endocytosis of the enucleated MSCs undergoing intrinsic apoptosis after nucleus enucleation, mediates the generation of CAR-M for glioblastoma therapy. (E) Engineered CAR-M loading bioactive HβCD can serve as living factory for lipid decomposition and the clearance of apoptotic cells in the inflammatory sites. (F) LNT cancer cells can serve as both delivery vehicles and cellular vaccines for transporting OVs in cancer immunotherapy.

Over the past three decades, researchers have actively explored the use of cells as drug delivery vehicles, as well as employing the cells themselves as therapeutic agents in immunotherapy, known as engineered cell therapies. Compared with nanoscale organelle-derived carriers, micro-sized active cell carriers and engineered cell drugs exhibit superior precision and efficacy in targeted delivery, owing to intrinsic properties such as cytokine-mediated chemotaxis, stem cell tumor tropism, inflammation-targeted homing, and platelet-mediated aggregation in response to tissue injury. In addition, engineered cell carriers can secrete cytokines and other bioactive molecules to modulate the immune microenvironment, thereby enhancing the therapeutic effects of immunomodulatory drugs. Biomacromolecular therapeutics can be incorporated via genetic engineering, overcoming the challenges associated with inefficient loading into inert carriers. Considering cell source and type, commonly employed platforms for drug delivery include blood cells, immune cells such as CAR-T cells and macrophages, bone marrow- or iPSC-derived MSCs and NSCs, as well as other cell types including bacteria and cancer cells (Fig. 4). Compared with nanoscale organelle-derived carriers, microscale engineered cell vehicles offer distinct advantages in preparation simplicity, drug loading diversity, genetic engineering for regulated drug release, scalability, and consistency of biological preparations (Table 4).

**Table 4:** Cell-based microscale BDDS for immunotherapy.

Cell types	Therapeutic cargos	Loading methods or engineering technologies	Main characteristics	Therapeutic applications in disease	Limitations	Clinical stage	References
**RBCs**	αPD-1	Sortase-mediated reaction	Enhancing the retention of RBCs in spleen and activation of T cells	Cancer patients with resistance to ICB therapy	Lacking tumor targeting	Phase I	[116]
	E6 peptide	Maleimide-thiol reaction	Stimulating the immune system	MC38 subcutaneous tumor	Risk of immune activation	Preclinical stage	[117]
**Platelets**	αPD-L1	Maleimide-thiol reaction	Targeting the wound and inflammation sites	Post-surgical cancer recurrence	Quality control	Preclinical stage	[118]
	αPD-1	Maleimide-thiol reaction, T cell-platelet conjugate	Bone marrow-targeted delivery and local release	Leukemia	Quality control	Phase I	[119]
	Granzyme B and perforin	Polyphenol-membrane protein interaction	T cells-mimicking to release the cytotoxic substances	Pulmonary metastasis of breast cancer and melanoma	Complicated preparation process	Preclinical stage	[120]
	OVs	Receptor-mediated endocytosis	CTCs-targeted OVs delivery	Pulmonary metastasis of various cancers	Risk of immune activation	Preclinical stage	[121]
	PROTACs for PD-L1	Ligand-mediated covalent labelling	Cancer-targeted PROTACs delivery for the intracellular or extracellular TPD	Cancer recurrence and metastasis	Potential off-target effects for tissue/cell	Preclinical stage	[122]
**Megakaryocytes**	OVs	Receptor-mediated endocytosis	*In vivo* production of oncolytic platelets	Post-surgical recurrence and pulmonary metastasis of cancers	Scale production	Preclinical stage	[123]
**ADSCs**	CD44 antibody-modified hydroxyapatite nanorods	Antigen-antibody biochemical reactions	The inherent tumor tropism and the osteogenic differentiation of ADSCs	Tumor and bone regeneration	Complicated preparation process and quality control	Preclinical stage	[124]
**MSCs**	Nintedanib	Receptor-ligand recognition of CD44 receptor and A6 peptide	Homing effect of MSCs for pulmonary fibrosis, MSCs differentiated into AEC II for lung injury repair	Pulmonary fibrosis	Complicated preparation process and quality control	Preclinical stage	[125]
	CAR-encoding plasmids	Cell enucleation	Macrophage-specific endocytosis of the enucleated cells undergoing intrinsic apoptosis, tumor-homing capability	Glioblastoma	Scale production	Preclinical stage	[126]
	IL-4 mRNA	Cell enucleation	Targeting inflammation sites and cytokine secretion abilities	Wound healing	Low drug loading and scale production	Preclinical stage	[127]
**Macrophages**	Anti-HER2 antibody	Genetic engineering modification	Immune cell recruitment, enhanced immune cell activity	Solid tumor	Limited tumor indications	Phase I	[128]
	HβCD	Genetic engineering modification	Engineered CAR-M as living factory for lipids decomposition and the clearance of apoptotic cells	Atherosclerosis	Complicated preparation process and quality control	Preclinical stage	[129]
	Attenuated bacterial vaccines (VNP20009)	Phagocytosis, LNT	Trojan horse-like cell carriers for the delay release and clearance of bacteria	Hepatocellular carcinoma	Potential risk of immune activation	Preclinical stage	[130]
**Neutrophils**	Cabazitaxel, teriparatide	Neutrophil-mediated phagocytic uptake	Aged neutrophils homing to the bone marrow	Bone metastasis cancer, osteoporosis	Complicated preparation process and quality control	Preclinical stage	[131]
	Drug-free polymer micropatches	Thiol-maleimide click reaction, receptor-ligand recognition	Micropatch-loaded neutrophils activate the systemic antitumor immune responses	Orthotopic breast cancer and melanoma	Scale production and 1uality control	Preclinical stage	[132]
**Mast cells**	OVs	Receptor-mediated endocytosis	Tumor antigen specific recognition activates therapeutics release	Various tumors	Potential risk of immune activation	Preclinical stage	[54]
**CAR-T cells**	IL-2, IL-10	Genetic engineering modification	Augmented proliferation and anti-tumor killing activity of the armored CAR-T cells	Cancer recurrence and metastasis	Potential risk of immune activation	Preclinical stage	[133,134]
	OVs	Antigen-receptor interaction	Intrinsic tumor tropism of CAR-T cells for cancer-targeted OVs delivery	Various cancers	Scale production and 1uality control	Preclinical stage	[135]
	CD19-targeted and BMCA-targeted CARs	Genetic engineering modification	Allogeneic T cells with low immunogenicity to eradicate pathogenic clones	SLE	Potential off-target effects	Phase I	[136,137].
**Cancer cells**	DOX	LNT	Cell vaccines/carriers	Acute myeloid leukemia (AML)	Potential safety risks	Preclinical stage	[36]
	ELE	Chemotherapeutic inactivation	Cell vaccines	Post-surgical cancer recurrence	Potential safety risks	Preclinical stage	[39]
	DOX/αPD-1	LNT	Pulmonary preferred delivery	Pulmonary metastasis of TNBC	Complicated preparation process and quality control	Preclinical stage	[138]
	cGAMP	Cell enucleation	Cell vaccines/carriers	Melanoma	Potential safety risks	Preclinical stage	[139]
	OVs	LNT	Cell vaccines/carriers	Lung cancer and glioblastoma	Potential safety risks	Preclinical stage	[140]
**Bacteria**	Bacterial vaccines (*E. coli* MG1655)	Engineered attenuation	Bacteria vaccines	Melanoma and hepatocellular carcinoma	Potential safety risks	Preclinical stage	[141]
	Antibiotics	UV irradiation	Drug vehicles for intra-biofilm drug delivery	Long-term immunity against implant infections	Scale production and 1uality control	Preclinical stage	[142]
	ICG	Photothermal elimination the activity of bacteria	Trojan bacteria platform for drug delivery across BBB	Glioblastoma	Potential safety risks	Preclinical stage	[143]

Platelets and RBCs, two of the most abundant blood cells, share structural features such as enucleation and participation in immune regulation. Platelets possess a short lifespan, small size, and intrinsic homing ability toward sites of vascular injury and inflammation, making them attractive vehicles for targeted immunotherapeutic drug delivery. Hu and Gu et al. systematically reviewed platform technologies, modification strategies, and clinical challenges of engineered platelets for tumor therapy [144]. Multiple platelet-drug conjugates (PDCs) have also been developed, including platelet-conjugated monoclonal antibodies P-αPD-1/P-αPD-L1, and platelet-conjugated granzyme B and perforin nanocomplexes, leveraging platelet availability, biocompatibility, and activation-dependent release of platelet-derived microparticles (PMPs) for targeted therapy [[118], [119], [120]]. Engineered platelets (eDePLTs) loaded with ligand-labeled HSP90 served as extracellular PD-L1 degraders to elicit potent antitumor immune responses in a triple-negative breast cancer mouse model [122]. Similarly, platelets were also applied to deliver OVs into CTCs for metastasis therapy [121]. Cryo-shocked platelet-liposome conjugates (CPSAT) via host-guest interactions were developed to achieve thrombus-targeted delivery of tissue plasminogen activator (tPA) and aspirin for thromboembolic diseases [145]. Besides blood cells, immune cells can not only act as drug carriers but also directly participate in regulating the body's immune system.

Neutrophils exhibit excellent inflammation-targeting ability and can release their contents including cytokines and ROS, to prevent the spread and expansion of pathogens in the apoptosis form of NETs. The characteristics make them ideal carriers for inflammatory-related diseases such as rheumatoid arthritis, pneumonia, ischemia-reperfusion injury, and neuroinflammation-related neurodegenerative diseases [146]. Utilizing neutrophils’ phagocytic capacity and natural trafficking across the bone marrow-blood barrier, the engineered neutrophils encapsulating cabazitaxel/teriparatide-loaded PLGA nanoparticles were generated for bone metastasis and osteoporosis immunotherapy [131]. Antibody-modified nanoparticles, such as CD11b-targeted drugs, can hijack neutrophils for targeted anti-inflammatory delivery in acute lung injury and other inflammatory conditions [147]. Neutrophils similarly serve as carriers across biological barriers such as BBB, enabling inflammation-responsive drug release in postoperative tumor sites for glioma therapy [148,149]. Polarized tumor-associated neutrophils have been leveraged for anti-CD11b-functionalized micropatch delivery in cancer immunotherapy [132]. Although neutrophil-based delivery systems exploit the remarkable chemotactic migration and rapid inflammatory recruitment capacity of neutrophils, their short lifespan and limited functional persistence restrict their applications in long-term disease modulation. In contrast, macrophages represent a more versatile cellular platform owing to their prolonged survival, extensive tissue residency, phagocytic activity, and remarkable phenotypic plasticity.

As key phagocytes, macrophages mediate pathogen clearance, antigen presentation, cytokine secretion, and tissue repair. Harnessing this capacity, engineered HER2-targeted CAR-M have advanced to clinical trials [128]. Beyond cancer therapy, engineered CD47-targeted CAR-M platforms loading hydroxypropyl β-cyclodextrin (HβCD) lipid nanoparticles (CAR-M/HβCD LNP), have been employed to enhance lipid clearance and promote atherosclerosis therapy [129]. Utilizing the strong phagocytic ability of macrophages to carry bacterial vaccines, LNT macrophages could be engineered as “Trojan horses” to deliver an attenuated Salmonella typhimurium VNP20009 (LNT MACS/VNP) for tumor-targeted immunotherapy [130]. Macrophage-drug conjugates (MDCs) have also been engineered to transfer heavy-chain ferritin-fused antibody-drug conjugates (HFt-ADC) between macrophages and cancer cells for glioblastoma therapy [150]. While macrophage-based delivery systems provide powerful opportunities for regulating pathological immune microenvironments through their phagocytic capacity, cytokine secretion, and polarization plasticity, their therapeutic outcomes are often influenced by the complexity and heterogeneity of inflammatory niches. To achieve more comprehensive tissue repair and functional restoration, stem cell-based delivery platforms have emerged as a promising strategy for immunotherapy.

**Fig. 5:**
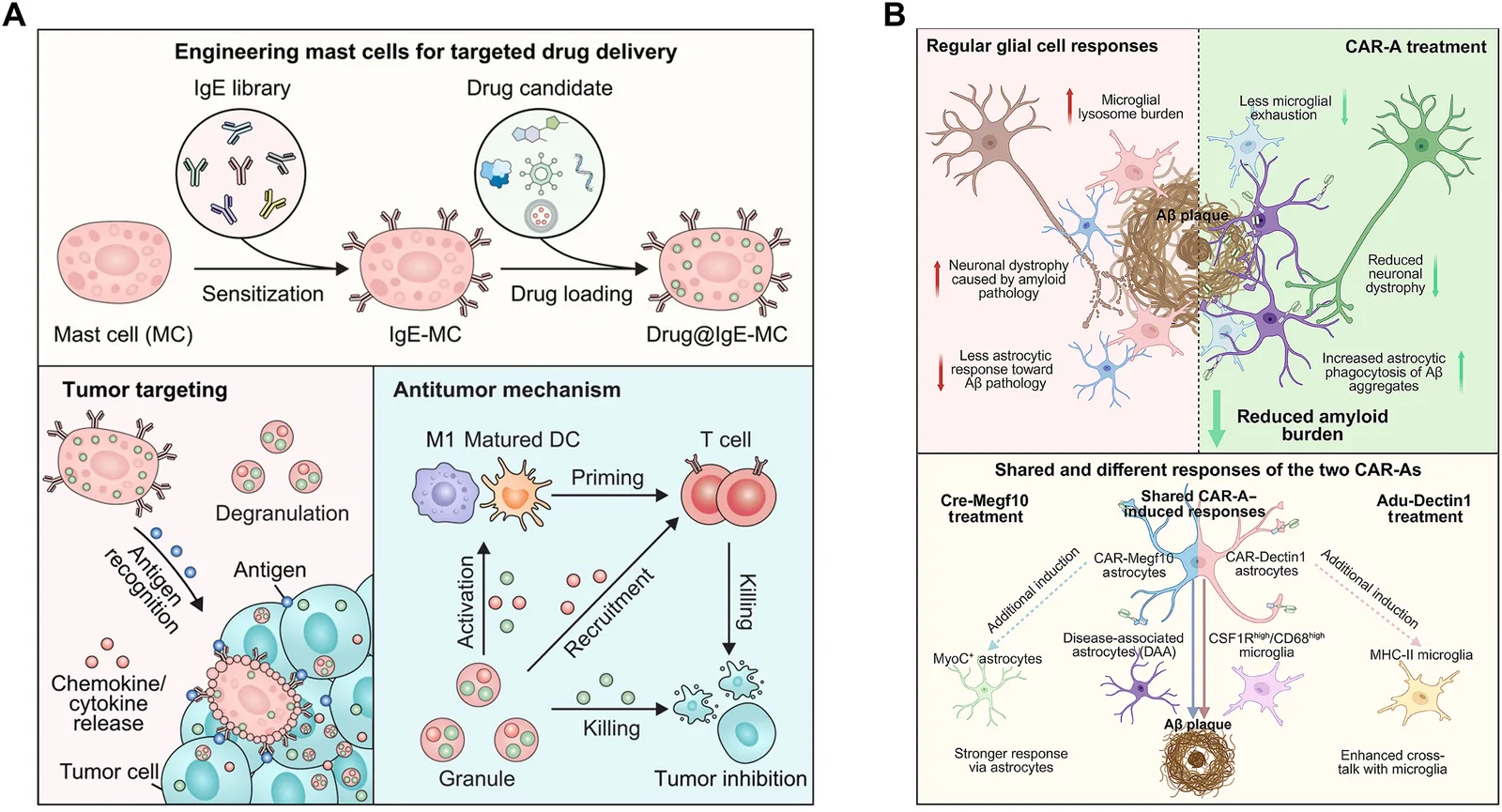
*In vitro and in vivo* programming cells as drug delivery vehicles or therapeutic cells for immunotherapy. (A) Engineered IgE-MCs are generated *in vitro* as a delivery platform to achieve the tumor-specific antigen-guided tumor-targeted drug delivery for cancer immunotherapy. *Reproduced with permission. Copyright 2026, Cell* [54]. (B) Astrocytes are engineered *in vivo* by AAV-PHP.eB-GFAP administration to *in situ* generate Aβ pathology-targeted CAR-A for long-term Aβ clearance over three months, to reduuce the nerve damage and alleviat neuroinflammation for AD treatments. *Reproduced with permission. Copyright 2026, Science* [155].

Representative immune cells such as T cells, NK cells and mast cells not only function as therapeutics, but also serve as vectors for anti-inflammatory and antitumor drugs. CAR-based cellular therapies have achieved clinical success, particularly CAR-T and CAR-NK products for hematological malignancies, while CAR-M therapies are entering early clinical evaluation for solid tumors. Strategies to enhance CAR-T infiltration and efficacy in solid tumors include expression of cytokines or receptors such as IL-2, IL-6, IL-8, and IL-10 [133,134,[151], [152], [153]]. To minimize off-target toxicity, CAR-T cells with organ-specific antigen recognition and tissue-targeted activation were engineered for controlled therapeutic cargo release [154]. Engineered oncolytic virus T cell chimeras (ONCOTECH) through pMHC-TCR interactions were developed to deliver PD-L1-encoding OVs (eOAs) for cancer immunotherapy [135]. CAR-T therapy is also being expanded to autoimmune diseases such as systemic lupus erythematosus (SLE). Engineered CAR-T cells *in vivo* targeting pathological B cells and long-lived plasma cells have demonstrated efficacy in phase I trials for systemic lupus erythematosus [136,137]. Mast cells were *in vitro* engineered as IgE-MCs for the tumor-specific antigen-guided tumor-targeted oncolytic virus delivery and local immune activation for cancer immunotherapy (Fig. 5A) [54]. Besides *in vivo* programming CAR-T for immunotherapy, amyloid-β (Aβ) pathology-targeted CAR-astrocytes (CAR-A) were generated by *in vivo* programming with noninvasive adeno-associated virus vehicle (AAV-PHP.eB-GFAP) administration for long-term Aβ clearance to delay the Alzheimer's disease (AD) progression in mice models (Fig. 5B) [155]. *In vitro* and *in vivo* programming immune cells or immunoregulatory cells as drug delivery vehicles or therapeutic cells, are becoming a mainstream area of research and widely applied in clinical immunotherapy.

Cancer cell and bacterium derived carriers have emerged as promising platforms for antitumor vaccines and immunotherapeutic drug delivery, provided that tumor cells are fully inactivated to eliminate pathogenicity. The dead triple-negative breast cancer (TNBC) cell platform via chemical conjugation with DOX-loaded liposomes and αPD-1, was developed to achieve lung-targeted drug delivery for chemo-immunotherapy of TNBC lung metastasis [138]. The enucleated cancer cells linked to cGAMP-loaded bacteria-derived vesicles could enhance vaccine immunogenicity and suppress melanoma progression [139]. OVs-loaded LNT cell platforms served as Trojan Horse-like, to facilitate the delivery of oncolytic adenovirus type 5/11 (Ad5/Ad11) and augment its immune response as tumor vaccine [140,156]. Bacterial platforms represent a unique class of biologically derived delivery systems, owing to their intrinsic tumor-homing capability, immune activation potential, and programmable genetic features. Unlike conventional synthetic carriers, certain bacteria can preferentially accumulate in hypoxic, necrotic, and immunosuppressive tumor regions, enabling localized delivery of therapeutic cargos. A bacteria-based supportive approach was developed that could boost the effectiveness of adoptive T cell therapy against solid tumors, by injecting the *E. coli* MG1655 strain directly into the tumor [141]. The inactivated bacteria also could deliver antibiotics across polymicrobial biofilms, to prevent implant-associated infections and elicit long-term immunity [142]. Beyond serving as drug delivery vehicles, bacteria can be engineered with photothermal components to generate localized heat and amplify antitumor immune responses. Furthermore, bacteria encapsulating photosensitive ICG-silicon nanoparticles have been developed as live antitumor vaccines capable of BBB penetration, and subsequent complete inactivation via photothermal effects for glioblastoma immunotherapy [143]. To ensure biosafety of these living cell carriers applied *in vivo*, various inactivation strategies including irradiation, ultraviolet sterilization, chemical inactivation, liquid nitrogen freezing, cell enucleation, and synthetic lethality strategies, have been explored and used to eliminate their pathogenicity.

Engineered cell carriers such as immune cells usually rely on innate chemotaxis for disease-specific accumulation. There are still some limitations associated with cross-species differences in inflammatory homing signals during clinical translation of engineered cell carriers. For example, neutrophil recruitment is regulated by conserved chemokine axes involving CXCR1/CXCR2 signaling, whereas important differences exist between human and murine systems. Human neutrophil recruitment depends strongly on CXCL8 signaling, whereas mice utilize alternative chemokine systems such as CXCL1/CXCL2. Similar species-dependent variations have been observed in macrophage and mast cell recruitment pathways, where differences in CCR/CXCR receptor expression and ligand affinity may influence tissue targeting efficiency. Therefore, preclinical evaluation of immune cell-based carriers should incorporate models that better recapitulate human inflammatory signaling networks, including humanized immune system models, patient-derived organoids, and *ex vivo* human immune cell migration assays. In parallel, engineering approaches that introduce synthetic chemokine receptors or programmable homing circuits, may improve the robustness and predictability of immune cell targeting across species to boost clinical translation of BDDS.

### 3.3 Macroscale: Tissue-derived and organoid platforms

Maintaining the bioactivity of macromolecular therapeutics, particularly cell-based drugs, remains a critical bottleneck for long-acting, sustained *in vivo* delivery. Artificially constructed physiological environments cannot fully replicate the optimal conditions for cell growth, survival, and functional activity. Organ or tissue transplantation is generally for the purpose of regeneration and repair. Human tissue-derived platforms may maximize the retention of cell-drug activity and support long-term survival, serving as both drug reservoirs and sustained-release vectors. Likewise, alternative organoids can function not only as therapeutic tissues but also as potential drug delivery carriers.

Organ and tissue transplantation has promoted the rapid development of tissue-like drugs and tissue-based platforms. Liver, islet, and skin transplantation have already been validated in mouse or pig models for the feasibility of drug secretion or delivery [157]. Apart from liver injury diseases, liver transplantation is acceptable to prolong median survival for hepatocellular carcinoma during ICB immunotherapy [158]. Islet transplantation, as a supplement to endogenous insulin, is a promising treatment for insulin-dependent type 1 diabetes mellitus (T1DM) over the past few decades [159]. The survival and functions of these transplanted tissues as well as living drugs are limited by the immune rejection and the insufficient vascularization *in vivo*. The continuous administration of immunosuppressive drugs is key to improving the success of organ or tissue transplantation in clinic. Allogeneic islet co-transplantation with the engineered immunomodulatory cells was explored in T1DM mice without immunosuppression [160]. The vascularization of human islets is a potential technology for the long-lasting and functional engraftment *in vivo* for T1DM treatment [161]. Clinical translation of tissue-derived therapies is progressing. Stanford Medicine's group successfully completed Phase III trials using genetically engineered skin to treat epidermolysis bullosa (EB) [162]. Engineered tissues, as an implantable living pharmacy, can produce multiple drugs including therapeutic proteins, enzymes and antibodies inside the body over the long term for immunotherapy.

**Fig. 6:**
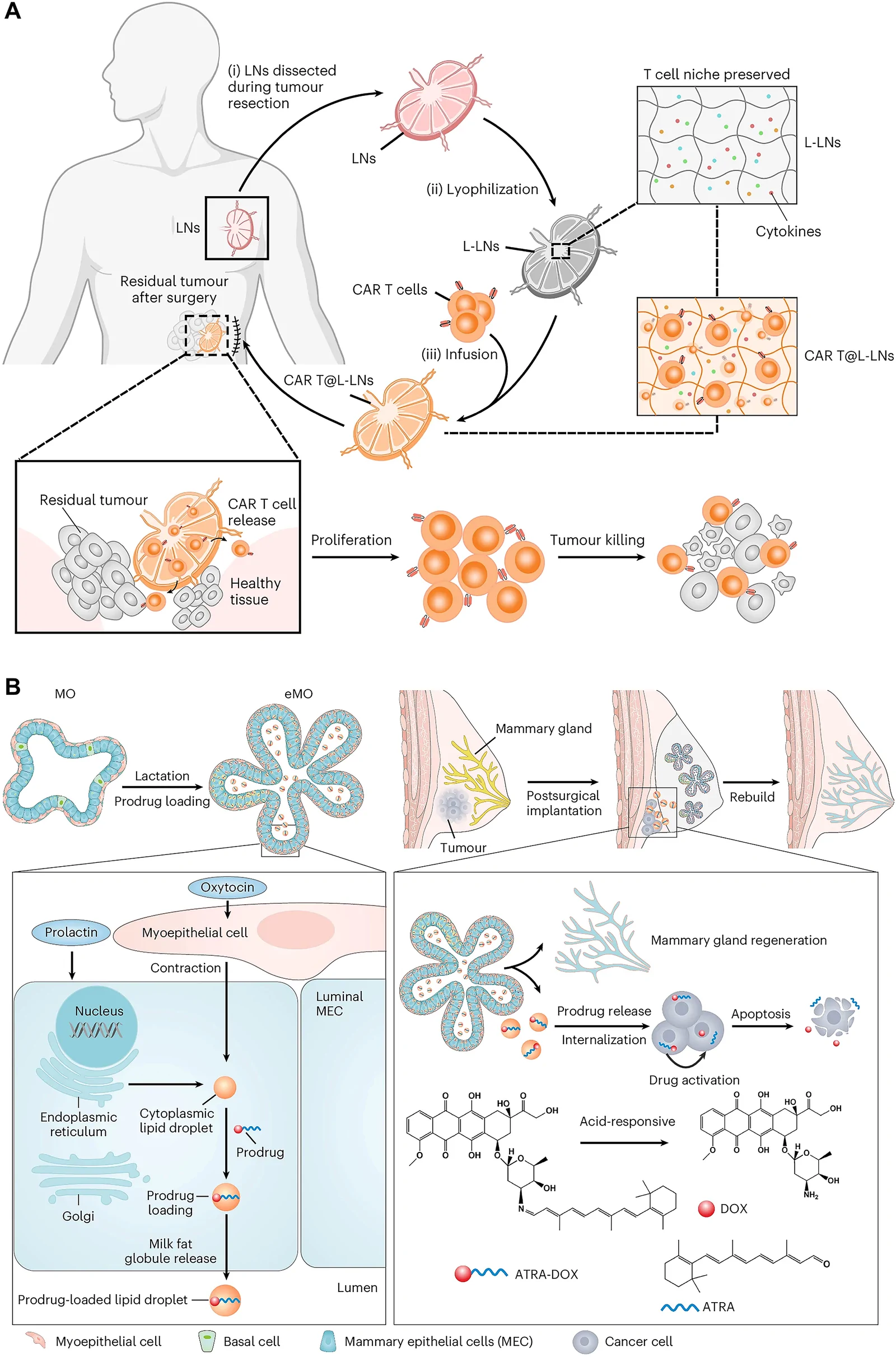
(A) Patient-derived frozen lymph nodes as a macroscale reservoir and delivery vehicle of CAR-T cells. The representative lymph node tissue can promote the *in vivo* proliferation, survival of and immune cytotoxic activity of CAR-T cells for antitumor immunotherapy. *Reproduced with permission. Copyright 2024,* Nature Materials [163]. (B) eMO as a macroscale drug secretion deport and the filler material for tissue repair and regeneration. Mammary organoid (MO) was induced into the lactation phenotype for antitumor prodrug secretion and transplanted in the postoperative excision of lesions for breast cancer chemotherapy. *Reproduced with permission. Copyright 2026,* Nature Biomedical Engineering [164].

Freeze-drying technology has initially addressed challenges associated with the storage and transport of biologically active macromolecular drugs. Recently, lyophilized lymph nodes were developed to enhance *in vivo* CAR-T cell delivery, leveraging the preserved tissue meshwork and abundant cytokine and chemokine content to promote efficient CAR-T cell loading, viability, and activation [163]. Clinically, tumor resections are often accompanied by lymph node dissection, providing a source of autologous tissue (Fig. 6A). Lymph nodes represent highly organized immune microenvironments containing extracellular matrix (ECM) networks, stromal compartments, and cytokine-binding niches that regulate immune cell survival, migration, and differentiation. During the freezing stage, water molecules form ice crystals, which reduce molecular mobility and limit structural rearrangements of ECM proteins and components that bind cytokines. Then, under low-pressure conditions, the ice crystals sublimate to remove water, forming a dry matrix while retaining the spatial organization of structural proteins, glycosaminoglycans (GAGs), and proteoglycan networks. These preserved molecular structures can maintain non-covalent interactions with cytokines and chemokines, including important T cell regulators like IL-7, IL-15, and CXCL19, thereby affecting T cell survival, proliferation, and migration. Freeze-drying preserves bioactive components while eliminating pathogenic risks, and the resultant loosened tissue structure facilitates the loading of cell-based therapeutics. Lyophilized tissue, as a promising vehicle for immunotherapy, is expected to address many issues such as drug loading, biocompatibility, and vascularization of transplanted tissues. Certainly, optimization of cryoprotectants, drying parameters, and post-rehydration functional validation remains essential to ensure preservation of immune-regulatory activity.

Organoids, as alternative tissue-derived carrier materials, can address the problem of insufficient sources of human tissues. Therapeutic organoid transplantation has already been investigted in multiple immune-related diseases. Human iPSC-derived skin organoids were demonstrated to alleviate early inflammation following frostbite and enhanced surface stem cell populations in murine models [165]. Human brain organoids that were integrated into the damaged visual cortex of adult rats, verified the functional neuronal responses to visual stimulation [166]. Similarly, iPSC-derived cerebral organoids transplanted into stroke model NOD-SCID mice could restore infarcted tissue and ameliorate sensorimotor deficits [167]. iPSC-derived liver organoids were generated to alleviate fibrosis through immunomodulatory effects in chemical fibrosis rat models upon transplantation onto fibrotic liver surfaces [168]. Organoids, serving as carriers for small-molecule drugs and specialized reservoirs for immunomodulatory agents, can directly facilitate or enhance interactions between immune cells and their target cells within the organoid microenvironments. Typically, the engineered mammary organoid (eMO) was developed as a repository of antitumor prodrugs, and induced activation to release chemotherapeutics in the form of milk secretion for the post-surgical chemotherapy against breast cancer (Fig. 6B) [164]. It was also demonstrated that the recipient mice could integrate the mammary organoids with the residual mammary tissue for post-surgical gland regeneration. At present, the quality standards for artificially cultured organoids applied to clinical translation are gradually being established for immunotherapy.

## 4 Emerging biosynthetic platforms

Biosynthetic platforms are usually defined as the engineered biological systems constructed from biomacromolecules, subcellular components, or living cells through biosynthesis, molecular assembly, and cell-based manufacturing approaches. By integrating biological building blocks across different organizational scales, these platforms enable the generation of higher-order functional systems, ranging from artificial organelles assembled from proteins and nucleic acids to engineered tissues and organoids generated through cellular organization and culture technologies. Biosynthetic platforms, including biomolecular condensates, artificial cells, organoids and artificial tissues, have emerged as complementary rather than competitive approaches to natural BDDS. Unlike naturally derived organelles, living cells and tissues, these biosynthetic platforms enable precise control over composition, physicochemical properties, and therapeutic payloads while minimizing donor-to-donor variability and manufacturing heterogeneity (Table 5). Compared with natural BDDS, the biosynthetic platforms offer superior programmability, modularity, scalability, and manufacturing consistency, facilitating standardized production and regulatory quality control. However, they generally lack the intrinsic biological complexity, active tissue homing, immune communication, and adaptive responsiveness naturally possessed by living cells or EVs. Consequently, rather than replacing natural BDDS, biosynthetic platforms are more likely to complement them by integrating synthetic controllability with biological functionality. Future hybrid systems combining natural biological components with programmable synthetic modules, may provide an effective strategy to maximize therapeutic efficacy while improving manufacturing reproducibility and clinical translation.

**Table 5:** Cross-scale comparison of emerging biosynthetic platforms.

Features	Biomolecular condensates	Artificial cells	Organoids and artificial tissues
Size scale	Nano-micro	Micro	Micro-macro
Mock object	Membraneless organelle	Intact cell	Tissue microenvironment
Biologicalcomplexity	Moderate	Low-moderate	High
Programmability	High	Very high	Moderate
Main cargo types	Proteins, genes, molecules	Proteins, genes, biologics	Cells, biologics
Targeting ability	Tunable	Engineerable	Tissue-specific
Manufacturing	Relatively simple	Complex	Highly complex
Preparation cost	Low	Moderate	High
Advantages	Scalable and quality-controllable	Multi-functional and structurally stable	Comprehensive immune microenvironment regulation
Challenges	Stability	Functional autonomy	Vascularization
Clinical maturity	Early	Early	Emerging
Immunogenicity	Low	Low-moderate	Moderate
Therapeutic applications in disease	Autoimmune diseases, microbial infection and cancers	Autoimmune diseases and cancers	Inflammation-mediated tissue injury

### 4.1 Membraneless organelle condensates

Inspired by membraneless organelles such as P-bodies observed in *Caenorhabditis elegans* by Brangwynne in 2009, the biosynthetic condensates have emerg as a novel vehicle for biological macromolecules delivery [169,170]. Condensates refer to membraneless compartments formed through liquid-liquid phase separation (LLPS) driven by multivalent biomolecular interactions. Biomacromolecules such as proteins, peptides, and nucleic acids, can form fluid microdroplets in salt ion buffer solutions, through hydrogen bonds, π-π interactions, cation-π interactions, electrostatic interactions, dipole interactions, and hydrophobic and hydrophilic interactions. The selective and highly efficient enrichment properties for biomacromolecules, enable condensates with excellent macromolecular drug loading capacity. The reversible formation and disassembly of condensates within cells, make it possible for drugs delivered into the cell to be released from the condensates. Therefore, biomolecular condensates induced by LLPS exhibit the potential applications for the delivery of nucleic acid drugs and protein/peptide molecules *in vitro* and *in vivo*. LLPS-driving condensates vesicles consisted of histone and cholesterol-modified DNA, were generated to deliver OVs for antitumor immunotherapy [171]. Peptide condensates coated by metal-phenolic membranes were also developed to encapsulate the immunoregulatory interferon alpha (IFN-α) for enhanced cancer immunotherapy via modulating glucose metabolism [172]. In fact, the microscale size and structural instability remain to be addressed for the potential application of biosynthetic condensates in immunotherapy.

### 4.2 Artificial cells

To mimick certain biologically celluar functions and avoid potential safety risks caused by the amplification, cancerization, and gene transmission of natural cells, researchers propose artificially constructing cell-like structures as synthetic bioreactors or delivery vectors. In general, artificial cells are synthetic systems composed of liposomes or polymer vesicles that provide external membrane and internal functional biological reaction systems such as organelle-like biosynthetic condensates, capable of carrying various bioactive macromolecules such as mRNA, enzymes, proteins. Artificial cells, as an emerging vehicles, exhibit the greatest advantage of designing functions according to needs. For example, controllable drug release, signal response, environmental perception, and metabolic reactions can be introduced artificial cell-based vehicles to make up for the limitations of natural cells in specific applications for immunotherapy. Artificial antigen-presenting cells (aAPCs), as a highly representative biosynthetic platform in cancer immunotherapy, can be generated via scalable manufacturing to achieve the consistency of its affinity and capture of antigen peptides, and cytokine release kinetics for precisely biological activity control [[173], [174], [175]]. How to achieve scalable production and precise *in vivo* targeting while ensuring safety, controllability, and functional stability, is the biggest bottleneck in the clinical translation of artificial cells in immunotherapy.

### 4.3 Advanced organoids and artificial tissues

Organoids derived from iPSCs that are generated from 3D culture systems, are limited in their size, due to the insufficient transport of nutrients and oxygen, and the inefficient clearance of cellular metabolic substances [176]. In addition, the organoids cultured *in vitro* only partially mimic the structure and function of organs. Chimeroids such as the chimeric brain organoids and vascularized organoids, as the advanced organoids, are expected to break through the existing bottlenecks in organoid culture *in vitro* and biomedical applications. The structural and functional integration of the advanced organoids with body tissues is the foundation of biomedical application of organoids. Brain chimeroids offer the opportunities for preclinical cell transplantation in organic brain syndrome and neurodevelopmental drug screening involving the cerebral cortex of children [177]. The emerging vascularized organoids boost organoid growth, maturation *in vitro*, and even promote their integration into body and prolong their lifespan *in vivo* [178]. Advanced organoids, while serving as drug carriers or reservoirs, can also integrate with the body's existing tissues to accelerate regenerative repair, and have good prospects for biomedical applications.

3D printing technology has promoted the development of cell printing-based artificial tissue biomedicine. A new printing system that can achieve both high cell density and maintain structural accuracy, has become a key demand in the field. Bioactivated biopriting method enables high cell-density artificial tissues with the complex organizational network architecture such as blood vessels and neural circuits [179]. Moreover, artificial tissues based on 3D cell printing are expected to address key issues such as low efficiency, standardization, and scalability in the production of tissue-derived drug delivery carriers, and even potentially provide alternative therapeutic tissues or organ transplantation for biomedical applications in terms of source and ethics.

## 5 Discussion and perspective

The selection of therapeutics for immune-related diseases is largely determined by disease characteristics and clinical manifestations, which consequently impose distinct requirements on BDDS in terms of size, physicochemical properties, and potential genetic engineering strategies. In cancer immunotherapy, small-molecule agents and monoclonal antibodies often suffer from off-target toxicity due to insufficient targeting, while engineered immune cells face challenges such as overcoming the immunosuppressive tumor microenvironment, sustaining viability, maintaining cytotoxic activity, and infiltrating solid tumors. In contrast, autoimmune diseases characterized by immune dysfunction frequently necessitate local administration of bioactive nutrients, cytokines, or engineered stem cells to achieve rapid and effective immunomodulation. Limited by BBB, the treatment of neuroinflammation generally prioritizes small-molecule anti-inflammatory drugs with BBB permeability or cell-based therapeutics that can cross BBB. Consequently, BDDS must be carefully designed to ensure precise targeting, controlled and sustained release, and the preservation of immune cell function, thereby optimizing therapeutic outcomes.

The therapeutic performance of BDDS is also strongly influenced by the administration routes, which can determine biodistribution, tissue accessibility, immune exposure, and clinical feasibility. Intravenous administration provides systemic distribution and is suitable for carriers requiring access to circulating immune cells or metastatic lesions, including EVs, engineered immune cells, and nanoparticles. However, the rapid clearance by the mononuclear phagocyte system and potential systemic immune activation remains major limitations. Local administration, including intratumoral, intra-articular, and intrathecal injection, enables high local concentrations and reduced systemic toxicity, making it attractive for solid tumors, autoimmune inflammation, and localized neurological disorders. Correspondingly, limited tissue penetration and invasive procedures restrict broader application. Intranasal delivery that provides a non-invasive route for crossing BBB, is particularly promising for neuroinflammatory diseases using exosomes, immune cell-derived vesicles, or cytokine carriers. Nevertheless, mucus clearance and variable absorption remain challenges. Oral administration offers excellent patient compliance but requires carriers with high gastrointestinal stability and epithelial transport capability. Current applications remain limited but may benefit from engineered bacteria and bioinspired oral delivery systems. Therefore, rational selection of administration routes should consider carrier characteristics, disease location, immune mechanisms, and clinical practicality.

The hierarchical design of BDDS from organoids and tissues to individual cells and organelles, directly governs drug-target interactions and therapeutic efficacy. Lower-level carriers primarily facilitate targeted delivery and controlled release, while higher-level architectures remodel local microenvironments to recruit and potentiate therapeutic cells. Instead of functioning as isolated therapeutic platforms, BDDS at different biological scales may be integrated to achieve complementary therapeutic functions. Future immunotherapies are expected to increasingly adopt cross-scale delivery strategies, in which nanoscale organelles, micro-size cellular carriers, and macroscale tissue-engineered constructs operate synergistically to overcome the multiple biological barriers encountered in drug delivery. For example, nanoscale EVs may be administered prior to microscale cell-based therapies to remodel immunosuppressive microenvironments by delivering immunomodulatory proteins or nucleic acids, thereby enhancing the subsequent infiltration, persistence, and antitumor activity of macrophages, dendritic cells, or engineered T cells. Likewise, mitochondria delivered by EVs or cell-derived vesicles could improve cellular bioenergetics and metabolic fitness before adoptive immune-cell transfer, potentially enhancing therapeutic durability. At the tissue level, macroscale engineered organoids or biomimetic tissues may serve not only as transplantation materials but also as programmable living factories capable of continuously producing therapeutic nanoscale EVs, cytokines, antibodies, or microscale immune cells *in situ*. Such tissue platforms could further be integrated with organelle transplantation or engineered cellular carriers to establish self-sustaining therapeutic systems with prolonged efficacy. Therefore, the convergence of multiple biological scales represents an important future direction for BDDS, to break through the drug delivery limitations of single administration route. Rather than considering organelles, cells, and tissues as independent delivery vehicles, the rational integration of their complementary biological functions may substantially improve targeting precision, therapeutic persistence, and immune regulation while reducing systemic toxicity. Developing standardized manufacturing processes, scalable production methods, and integrated biosafety evaluation frameworks, will be essential for translating these cross-scale immunotherapeutic platforms into clinical applications.

Future advances in BDDS will depend not only on the discovery of new biological carriers, but also on the integration of emerging computational and engineering technologies that accelerate platform optimization and clinical translation. Artificial intelligence (AI) and machine learning (ML) are expected to play increasingly important roles in predicting carrier-cargo compatibility, optimizing donor selection, identifying critical quality attributes, and modeling biodistribution, immune interactions, and therapeutic efficacy from multidimensional datasets. AI/ML-assisted analysis of multi-omics and imaging data may further facilitate the rational design of BDDS with improved targeting specificity, biological functionality, and manufacturing consistency. In the short term, AI-based models can integrate multi-omics datasets, proteomic profiles, membrane compositions, and physicochemical parameters to predict key carrier properties, including cellular targeting capability, biodistribution, immune compatibility, cargo-loading efficiency. For EVs and membrane-derived carriers, deep-learning algorithms combined with single-cell sequencing and proteomics may identify functional surface markers associated with tissue homing and immune regulation, enabling the rational selection or engineering of donor cells. In engineered cellular systems, computational modeling can facilitate the optimization of synthetic gene circuits, receptor expression levels, and metabolic states to improve therapeutic persistence and safety. For biosynthetic platforms such as condensates and artificial cells, molecular simulation and AI-guided protein design may accelerate the discovery of programmable interactions controlling cargo encapsulation and release. Meanwhile, high-throughput microfluidic technologies provide powerful platforms for standardized and scalable production of biologically derived carriers while enabling rapid screening of formulation parameters, encapsulation efficiency, membrane engineering strategies, and functional performance. In the short term, microfluidic platforms can be applied to the standardized EVs isolation, organelle-derived platforms engineering, controlled assembly of artificial cells, and rapid formulation screening. For example, microfluidic chips integrating size-based separation, immunoaffinity capture, and automated characterization can improve EVs purification efficiency while reducing batch-to-batch variation. Coupling automated microfluidic manufacturing with real-time quality monitoring and robotic workflows could substantially improve production reproducibility while reducing labor-intensive operations. For engineered cells and organoid-derived platforms, automated microfluidic bioreactors may enable precise regulation of nutrient exchange, oxygen supply, cell differentiation, and maturation states. In the long term, integration of microfluidics with AI-driven feedback systems may establish autonomous manufacturing platforms capable of real-time monitoring and optimization of BDDS production under GMP-compatible conditions. Such interdisciplinary convergence will be instrumental in overcoming current throughput limitations and accelerating the clinical translation of next-generation biologically derived therapeutics.

Despite remarkable advances in BDDS, their successful clinical translation remains limited by challenges associated with manufacturing consistency, regulatory complexity, safety evaluation, and economic feasibility. Unlike conventional synthetic carriers, BDDS span a broad spectrum from acellular carriers including isolated organelles, membrane-based carriers and condensates to living therapeutic systems such as engineered cells and organoids, creating distinct regulatory challenges. Nanosacle organelle isolation and large-scale production remain inefficient, and challenges such as heterogeneity, low drug-loading capacity, and immature targeting technologies persist. Microscale carriers, including engineered immune and stem cells, increasingly integrate genetic modifications to enhance therapeutic efficacy in cancer, autoimmune, and neuro-immune disorders, yet obstacles in cell sourcing, *in vitro* expansion, genetic engineering, and post-transplant survival remain. At the macroscale, tissue-derived carriers such as islets, lymph nodes and organoids provide physiologically relevant microenvironments rich in cytokines and nutrients, supporting *in situ* cell activation and sustained drug release. Artificially cultured organoids are gaining traction as drug carriers, though issues of size, vascularization, and functional integration continue to challenge their clinical translation. Compared with normal synthetic platforms, the major translational challenges of living BDDS are the inherent biological variability of donor origin cells or tissues, long-term immunogenicity and tumorigenicity, batch-to-batch heterogeneity caused by scalable GMP manufacturing. Given the individual and species differences in material sources, reliable preclinical evaluation of BDDS requires appropriate selection of animal models and rigorous experimental design. Rather than immunocompetent and immunodeficient mouse models, humanized mouse models partially overcome these limitations such as differences between murine and human immune systems and the absence or severe impairment of immune components, by introducing human immune cells or hematopoietic stem cells into immunodeficient backgrounds. Future BDDS studies may further benefit from integrating human organoids, patient-derived models, and multi-component immune systems to bridge the gap between animal experiments and clinical trials. Therefore, the scalable production strategies, universal donor sources, standardized manufacturing pipelines, real-time quality and biosafety monitoring and cost-effectiveness analyses will be necessary to establish competitive advantages over synthetic alternatives. To accelerate clinical implementation, BDDS development should follow a staged translational roadmap: Stage I, platform optimization and standardization (0-3 years); Stage II, preclinical validation and regulatory alignment (3-5 years); Stage III, clinical translation and commercialization (5-10 years). Prioritize disease indications with strong unmet medical needs and favorable risk-benefit profiles, including cancer immunotherapy, autoimmune diseases, inflammatory disorders, and regenerative immunology. Integration with AI-driven design, automated manufacturing, and precision medicine approaches will further accelerate the development of next-generation BDDS platforms. Collectively, the future success of BDDS will depend not only on demonstrating biological efficacy, but also on achieving manufacturing robustness, regulatory acceptance, long-term safety, and economic sustainability.

## CRediT authorship contribution statement

**Xi Tan:** Conceptualization, Data curation, Investigation, Methodology, Writing – original draft. **Mutang Li:** Data curation, Investigation. **Dandan Luo:** Data curation, Investigation, Methodology. **Hongjun Li:** Conceptualization, Funding acquisition, Supervision, Writing – review & editing.

## Ethics

This is a review article. The research was not involve in any biological samples or animal experiments.

## Declaration of competing interests

The authors declare that they have no competing financial interests or personal relationships that could have appeared to influence the work reported in this paper.

## Data Availability

No data was used for the research described in the article.
